# Supplementary material for: Rapid molecular testing or chest X-ray or tuberculin skin testing for household contact assessment of tuberculosis infection: A cluster-randomized trial
Source: PLoS Med. 2025 Jul 28;22(7):e1004666. doi: 10.1371/journal.pmed.1004666 (PMC12316388; doi:10.1371/journal.pmed.1004666)

**Supplemental Figure 1: Schematic of the three strategies**

**Figure S1a: SCHEMATIC OF STRATEGY 1: Standard** *(based on Algorithm in WHO LTBI guidelines 2018)*


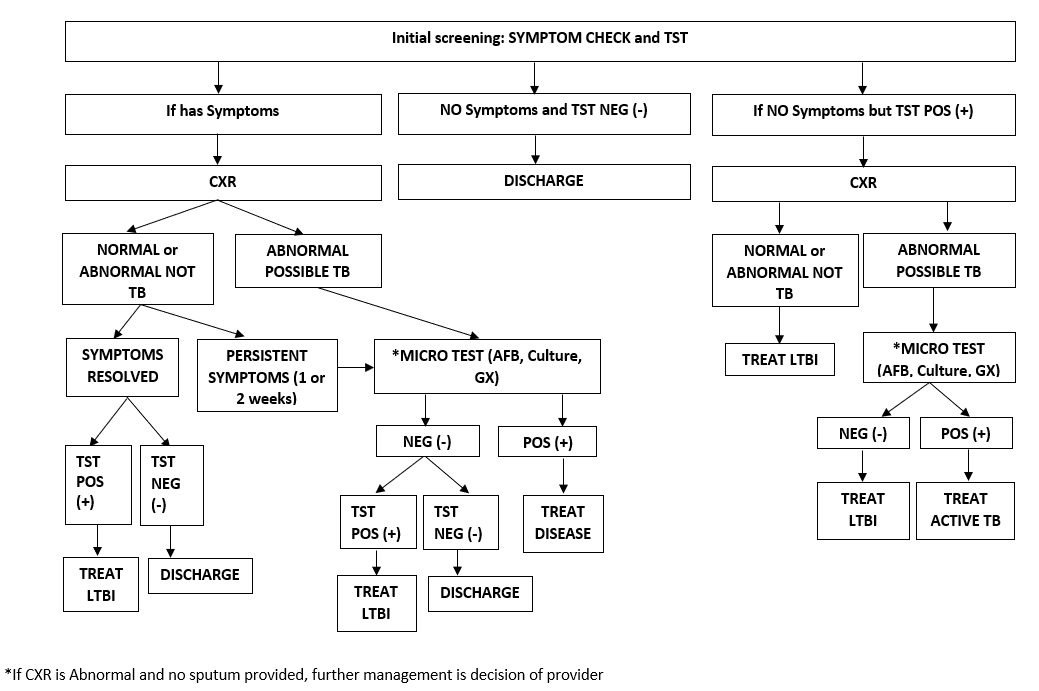


**Figure S1B: SCHEMATIC OF STRATEGY 2: Rapid Molecular Test replaces CXR**
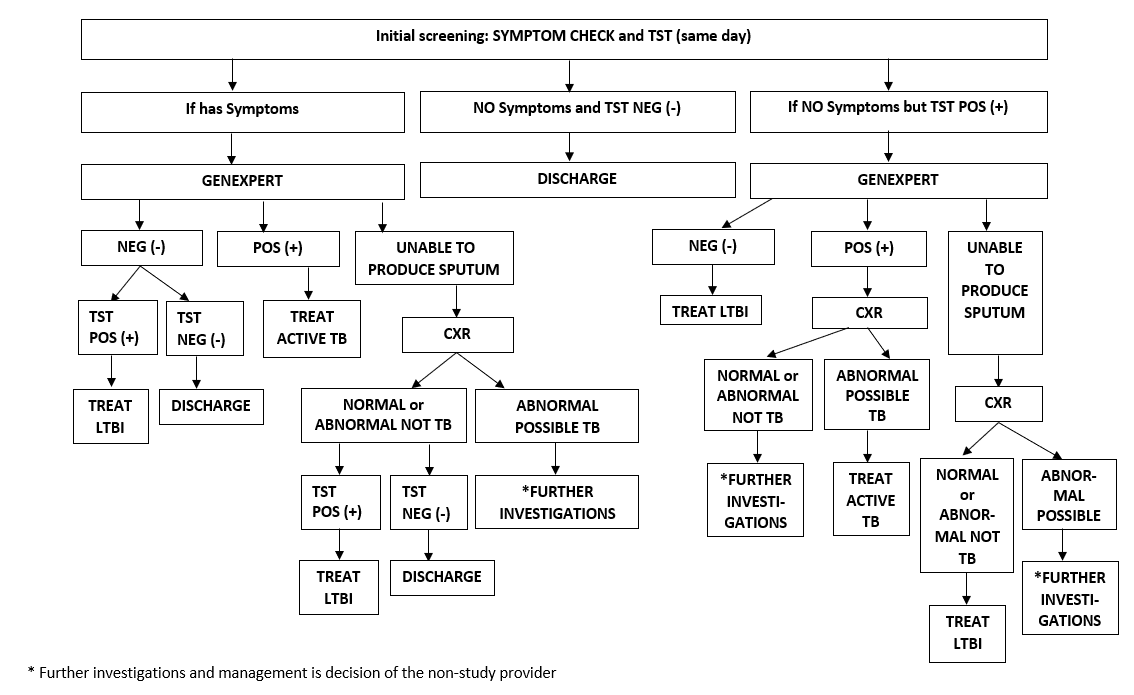


**Figure S1C: SCHEMATIC OF STRATEGY 3 (no-TST, Universal CXR)**


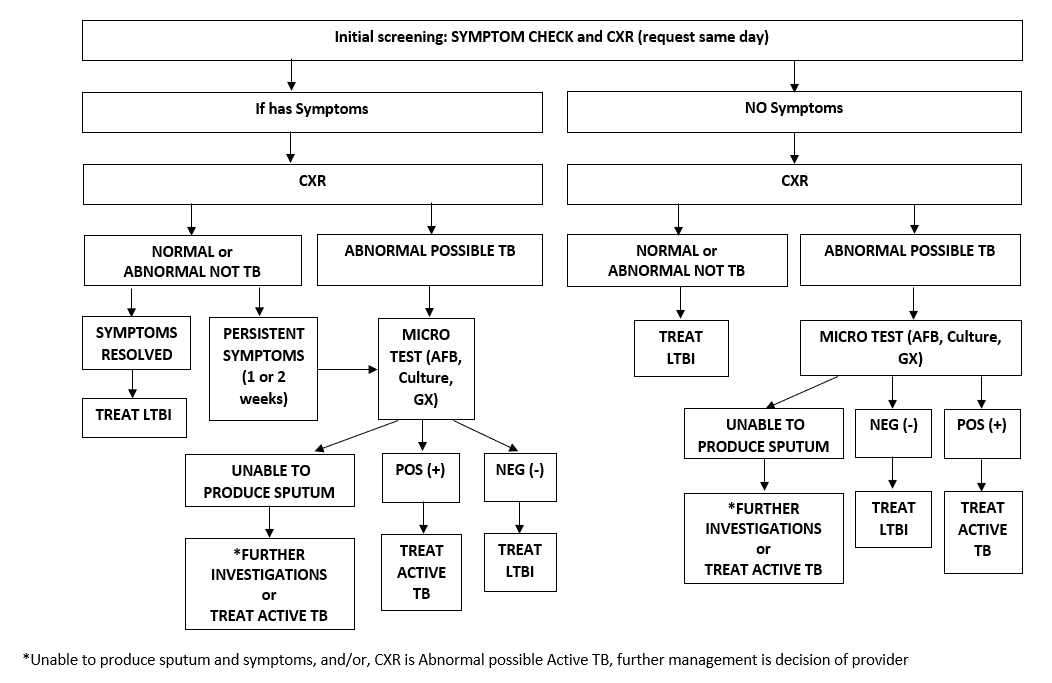

Supplement: S1 Fig — (DOCX) [file pmed.1004666.s001.docx]
