# Supplementary material for: Rapid molecular testing or chest X-ray or tuberculin skin testing for household contact assessment of tuberculosis infection: A cluster-randomized trial
Source: PLoS Med. 2025 Jul 28;22(7):e1004666. doi: 10.1371/journal.pmed.1004666 (PMC12316388; doi:10.1371/journal.pmed.1004666)
Supplement: S1 Tables — Table A: Summary of Index TB patients. Table B: Characteristics of HHC associated with Positive TST (among tested, in Strategies 1 and 2). Table C: Descriptive analysis of characteristics of participants who started and completed TPT. Table D: Unadjusted estimates of effect of incentives on outcomes. Table E: Adjusted risk differences in outcomes between experimental Strategies 2 and 3, and standard Strategy 1, stratified by receipt of incentives. Table F: Detailed health system costs in Benin and Brazil, in 2022 USD. Table G: Costs of drugs used for TPT, from 2022 GDF catalogue. Table H: Prevalence of positive TST stratified by country and age group, used to estimated number with TB infection (TBI) among HHC randomized to Strategy 3 (No-TST). (DOCX) [file pmed.1004666.s002.docx]

**Supplemental Tables**

**Table A: summary of Index TB patients**

| **Characteristic** | **Overall** | **Strategy 1**  **(Standard)** | **Strategy 2**  **(RMT)** | **Strategy 3**  **(No-TST)** |
| --- | --- | --- | --- | --- |
| Number of eligible index TB patients who participated | 459 | 149 | 155 | 155 |
| Benin | 327 | 105 | 112 | 110 |
| Brazil | 132 | 44 | 43 | 45 |
| Age of Index TB patients (N, %) |  |  |  |  |
| 12-14 | 8 (1.7) | 4 (2.7) | 2 (1.3) | 2 (1.3) |
| 15-24 | 109 (23.7) | 34 (22.8) | 33 (21.3) | 42 (27.1) |
| 25-34 | 111 (24.2) | 43 (28.9) | 38 (24.5) | 30 (19.4) |
| 35+ | 231 (50.3) | 68 (45.6) | 82 (52.9) | 81 (52.3) |
| Age (median [IQR]) | 35 [24, 47] | 32 [24, 48] | 35 [25, 49] | 35 [24, 46] |
| Number of Household contacts: |  |  |  |  |
| Overall median [IQR] | 3 [1, 5] | 3 [1, 4] | 3 [2, 5] | 3 [1, 5] |
| Overall mean [range] | 3.5 [1, 46] | 3.2 [1, 14] | 3.8 [1, 46] | 3.4 [1,12] |
| Benin mean [range] | 3.9 [1, 46] | 3.6 [1, 14] | 4.3 [1, 46] | 3.8 [1, 12] |
| Brazil mean [range] | 2.4 [1, 8] | 2.3 [1, 7] | 2.3 [1, 8] | 2.5 [1, 7] |
| Abbreviations:  RMT: Rapid molecular test (in this study GeneXpert MTB-Rif); No-TST: No Tuberculin skin test with Chest Xray for all HHC;  Notes: Information on index patients very limited. | | | | |

**Table B: Characteristics of HHC associated with Positive TST**

**(among tested, in Strategy 1 and 2)**

| **Characteristic** | **Total** | **Negative TST*** | **Positive TST*** |
| --- | --- | --- | --- |
| Total Number | 1021 | 596 | 425 |
| Strategy 1: Standard (n, %) | 457 (44.8) | 258 (43.3) | 199 (46.8) |
| Strategy 2: RMT (n, %) | 564 (55.2) | 338 (56.7) | 226 (53.2) |
| Age Group (n, %) |  |  |  |
| 5-14 | 413 (40.5) | 263 (44.1) | 150 (35.3) |
| 15-24 | 245 (24.0) | 144 (24.2) | 101 (23.8) |
| 25-34 | 174 (17.0) | 96 (16.1) | 78 (18.4) |
| 35+ | 189 (18.5) | 93 (15.6) | 96 (22.6) |
| Age in years (median [IQR]) | 18 [11, 29] | 16 [10, 27] | 20 [12, 32] |
| Male (n, %) | 493 (48.3) | 290 (48.7) | 203 (47.8) |
| Female (n, %) | 528 (51.7) | 306 (51.3) | 222 (52.2) |
| BMI (mean (SD) | 20.56 (6.01) | 20.02 (5.93) | 21.32 (6.05) |
| Smoking: never (n, %) | 977 (95.7) | 575 (96.5) | 402 (94.6) |
| Current or ex-smoker (n, %) | 44 (4.3) | 21 (3.5) | 23 (5.4) |
| Alcohol: never (n, %) | 695 (68.1) | 433 (72.7) | 262 (61.6) |
| Ever drank any amount (n, %) | 326 (31.9) | 163 (27.3) | 163 (38.4) |
| Abbreviations:  RMT: Rapid molecular test (in this study GeneXpert MTB-Rif); ; HHC: Household contact; TST: Tuberculin skin test; ;  Notes  *Positive TST includes those with prior TB disease or a positive TST (in Benin defined as >=10mm, and in Brazil as >=5mm) without prevalent TB disease. Negative TST defined as TST <10mm in Benin, and <5mm in Brazil. | | | |

**Table C: Descriptive analysis of characteristics of participants who started and completed TPT**

| **Variable** | **Started TPT*** | **Did not Start TPT** | **Completed TPT**** | **Did not Complete TPT** |
| --- | --- | --- | --- | --- |
| Characteristic |  |  |  |  |
| Total Number | 802 | 46 | 547 | 255 |
| Strategy 1: Standard (n, %) | 158 (95.2) | 8 (4.8) | 118 (74.7) | 40 (25.5) |
| Strategy 2: RMT (n, %) | 194 (94.2) | 12 (5.9) | 138 (71.1) | 56 (28.9) |
| Strategy 3: No-TST (n, %) | 450 (94.5) | 26 (5.5) | 291 (64.7) | 159 (35.3) |
| Age Group (n, %) |  |  |  |  |
| 5-14 | 331 (97.4) | 9 (2.6) | 241 (72.8) | 90 (27.2) |
| 15-24 | 201 (94.8) | 11 (5.2) | 130 (64.7) | 71 (35.3) |
| 25-34 | 121 (88.3) | 16 (11.7) | 70 (57.9) | 51 (42.1) |
| 35+ | 149 (93.7) | 10 (6.3) | 106 (71.1) | 43 (28.9) |
| Age (median [IQR]) | 17 [10, 29] | 26 [17, 32] | 16 [10, 29] | 19 [11, 29] |
| Male (n, %) | 389 (96.0) | 16 (4.0) | 270 (69.4) | 119 (30.6) |
| Female (n, %) | 413 (93.2) | 30 (6.8) | 277 (67.1) | 136 (32.9) |
| BMI (mean (SD)) | 20.4 (6.0) | 24.2 (6.3) | 20.2 (6.1) | 20.8 (5.8) |
| Smoking: never (n, %) | 774 (95.1) | 40 (4.9) | 531 (68.6) | 243 (31.4) |
| Current or ex-smoker (n, %) | 28 (82.4) | 6 (17.6) | 16 (57.1) | 12 (42.9) |
| Alcohol: never (n, %) | 530 (96.0) | 22 (4.0) | 368 (69.4) | 162 (30.6) |
| Ever drank any amount (n, %) | 272 (91.9) | 24 (8.1) | 179 (65.8) | 93 (34.2) |
| Abbreviations:  RMT: Rapid molecular test (in this study GeneXpert MTB-Rif); No-TST: No Tuberculin skin test with Chest Xray for all HHC; TPT: Tuberculosis preventive treatment; HHC: Household contact; TST: Tuberculin skin test; TBI: TB Infection; BMI: body mass index;  Notes  *Started TPT at any time after enrolment. Denominator is the number of HHC considered eligible for TPT.  ** Completed TPT in any amount of time, of all HHC recommended to start TPT. | | | | |

**Table D: Unadjusted estimates of effect of incentives on outcomes**

| **Outcome** | **Strategy 1**  **(Standard)** | | **Strategy 2**  **(RMT)** | | **Strategy 3**  **(No-TST)** | |
| --- | --- | --- | --- | --- | --- | --- |
|  | **With Incentives** | **Without Incentives** | **With Incentives** | **Without Incentives** | **With Incentives** | **Without Incentives** |
| N Participants | 197 | 277 | 238 | 345 | 230 | 302 |
| Completed Investigations* | 191 (97%) | 248 (90%) | 233 (98%) | 321 (93%) | 225 (98%) | 267 (88%) |
| Eligible for TPT** | 58 | 108 | 69 | 137 | 214 | 262 |
| Started TPT (% of eligible)** | 57 (98%) | 101 (94%) | 68 (99%) | 126 (92%) | 211 (99%) | 239 (91%) |
| Started TPT within 3 months (% of eligible)** | 57 (98%) | 96 (89%) | 68 (99%) | 123 (90%) | 211 (99%) | 238 (91%) |
| Completed TPT (% of started) | 38 (67%) | 80 (79%) | 43 (65%) | 95 (75%) | 142 (67%) | 149 (62%) |
| Completed TPT (% of eligible)** | 38 (66%) | 80 (74%) | 43 (64%) | 95 (69%) | 142 (66%) | 149 (57%) |
| Abbreviations:  RMT: Rapid molecular test (in this study GeneXpert MTB-Rif); No-TST: No Tuberculin skin test with Chest Xray for all HHC; TPT: Tuberculosis preventive treatment; HHC: Household contact; TST: Tuberculin skin test; TBI: TB Infection;  Notes  * Completed investigations mandated by the protocol, within 3 months of enrolment, of all HHC randomized to the strategy  ** In Strategies 1&2 eligible for TPT if TST positive and CXR normal and/or GeneXpert or other microbiologic tests negative in Strategies 1&2; In Strategy 3 eligible for TPT if CXR normal, and GeneXpert or other microbiologic tests negative if applicable. | | | | | | |

**Table E: Adjusted risk differences in outcomes between experimental Strategies 2 & 3, and standard Strategy 1, stratified by receipt of incentives**

*(Values shown are estimated differences in number of persons with outcome per 100 eligible.)*

| **Outcome** | **Strategy 2 vs 1** | **Strategy 3 vs 1** | **Strategy 3 vs 2** | **Strategy 3 vs 1&2** |
| --- | --- | --- | --- | --- |
| **Completed Investigations*** | |  |  |  |
| **With Incentives** | 0.8 (-4.9, 6.4) | 1.1 (-4.2, 6.4) | 0.3 (-3.7, 4.3) | 0.7 (-3, 4.4) |
| **No incentives** | 1.6 (-6.4, 9.6) | 1.2 (-7.7, 10) | -0.4 (-9, 8.2) | 0.4 (-7.4, 8.1) |
| **Started TPT (% of eligible)**** | |  |  |  |
| **With Incentives** | 0.2 (0, 0.3) | 0.2 (0, 0.4) | 0 (-0.1, 0.1) | 0.1 (0, 0.3) |
| **No incentives** | -5.6 (-13.2, 1.9) | -6 (-16.2, 4.2) | -0.4 (-11.1, 10.3) | -2.8 (-12.8, 7.2) |
| **Completed TPT (% of started) *** | |  |  |  |
| **With Incentives** | 4.9 (-18, 27.9) | -6.4 (-27.5, 14.7) | -11.3 (-30, 7.4) | -9.1 (-25.3, 7.1) |
| **No incentives** | 2.5 (-12.5, 17.4) | -15.2 (-30.2, -0.1) | -17.6 (-32.5, -2.8) | -16.4 (-29.4, -3.5) |
| Abbreviations: TPT: Tuberculosis preventive therapy; HHC: Household contact; TST: Tuberculin skin test;  Notes:  Adjusted with a Poisson regression model, using an identity link, and estimate via generalized estimating equations (GEE) adjusting for clustering. An exchangeable correlation structure and empirical standard errors were used.  *Adjusted for clustering by Index TB patient, and for HHC age, sex, country  ** Adjusted for clustering by site | | | | |

**Table F: Detailed health system costs in Benin and Brazil, in 2022 USD**

| Activities | **Benin** | | **Brazil** | |
| --- | --- | --- | --- | --- |
|  | Value | Reference* | Value | Reference* |
| **Costs for symptoms screen** | $3.25 |  | $5.46 |  |
| Nurse time, in minutes, to conduct symptoms screen | 7.50 | (1) | 7.50 | (1) |
| Nurse salary full time employee hourly | $3.51 | (2) | $12.77 | (3) |
| Cost per outpatient visit | $2.81 | (4) | $3.86 | (4) |
| **Costs for TST application** | $8.41 |  | $14.71 |  |
| Nurse time, in minutes, to place TST | 4.5 | (1) | 4.5 | (1) |
| Nurse salary full time employee hourly | $3.51 | (2) | $12.77 | (3) |
| Materials for TST | $5.34 | (5) | $9.89 | (6) |
| Cost per outpatient visit | $2.81 | (4) | $3.86 | (4) |
| **Costs for TST reading** | $2.98 |  | $4.48 |  |
| Nurse time to read TST | 2.9 | (1) | 2.9 | (1) |
| Nurse salary full time employee hourly | $3.51 | (2) | $12.77 | (3) |
| Cost per outpatient visit | $2.81 | (4) | $3.86 | (4) |
| **Acid fast bacilli** | $1.03 | (7) | $2.86 | (3) |
| **Mycobacterium TB culture^4^** | $7.85 |  | $3.82 | (3) |
| **Induced Sputum** | - |  | $53.47 |  |
| Technician time, in minutes, to perform test | - |  | 15 | Assumed**^2^** |
| Technician salary full time employee hourly | - |  | $12.77 | (3) |
| Lab fees | - |  | $46.41 | Extrapolated^3^ |
| Cost per outpatient visit | - |  | $3.86 | (4) |
| **Costs GeneXpert** | $21.82 | (2) | $17.38 | (3) |
| **Costs Chest X ray** | $31.21 | (5) | $7.93 | (3) |
| **Costs medical evaluation** | $3.99 |  | 7.56 |  |
| Physician time, in minutes, to conduct medical evaluation | 9.7 | (1) | 9.7 | (1) |
| Physician salary full time employee hourly | 7.3 | (2) | $22.87 | (3) |
| Cost per outpatient visit | $2.81 | (4) | $3.86 | (4) |
| **ALT & AST** | $11.18 | (7) | $2.74 | (3) |
| **Bilirubin** | $10.05 | (7) | $1.33 | (3) |
| **Hemogram** | $7.84 | (7) | $2.80 | (3) |
| **HIV test** | $2.26 | (5) | $6.80 | (3) |
| **Serum c reactive protein^4^** | $5.49 |  | - |  |
| **Serum creatinine^4^** | $3.14 |  | $1.26 | (3) |
| **Serum urea^4^** | $3.14 |  | $1.22 | (3) |
| **Serum magnesium** | - |  | $1.33 | (3) |
| **Serum TSH** | - |  | $5.91 | (3) |
| **Serum B12** | - |  | $10.04 | (3) |
| **Serum T4** | - |  | $7.65 | (3) |
| **Serum vitamin D** | - |  | $10.05 | (3) |
| **Urinalysis** | - |  | $2.44 | (3) |
| **Serum glucose** | - |  | $1.34 | (3) |
| **Gama GT** | - |  | $2.39 | (3) |
| **Malaria test^4^** | $1.57 |  | - |  |
| **Costs for follow up visits in the facility** | $3.60 | (4) | $6.34 |  |
| Time physician, in minutes to conduct FU visit | $6.5 | (1) | 6.5 | (1) |
| Physician salary full time employee hourly | $7.30 | (2) | $22.87 | (3) |
| Cost per outpatient visit | $2.81 | (4) | $3.86 | (4) |
| **Costs for follow up visits at home** | $8.40 | (2) | $14.77 | Extrapolated^5^ |
| **Costs to manage AE (same as FU visits)** | - |  | $6.34 |  |
| **Medications to manage AE** |  |  |  |  |
| Butylscopolamine per pill | - |  | $0.22 | (8) |
| Orphenadrine with dipyrone | - |  | $0.34 | (8) |
| Dexamethasone | - |  | $0.12 | (8) |
|  |  |  |  |  |
| Notes:  1-“-“ denotes a test or activity not performed in the country during the study.  2-There was no published information on the time required to conduct induced sputum; however, local staff informed us that it takes about 15 minutes to perform the test.  3-Additional fees were applied for managing the sample and additional materials. Since we did not have this information from the Brazilian sites, we extrapolated from the costs of induced sputum conducted in Canada. We used the value of AFB in Canada and calculated the ratio (i.e., Costs of induced sputum in Canada/Costs of AFB in Canada), which was 3.6. We then multiplied this ratio by the costs of AFB in Brazil.  4. Direct communication with the site investigator provided unit costs collected from *the Centre National Hospitalier Universitaire de Pneumo-Phtisiologie de Cotonou* where the study took place  5-There was no data on the cost of a home visit in Brazil, so we extrapolated from Benin, where the cost was 2.33 times higher compared to visits conducted in the facility. | | | | |

**Table G: Costs of drugs used for TPT, from 2022 GDF catalogue.**

| **Drug** | Unit costs per dose |
| --- | --- |
| **Isoniazid** |  |
| 100 to 250 mg | 0.02 |
| 300 to 500 mg | 0.02 |
| 600 to 900 mg | 0.05 |
| **Rifampin** |  |
| 150 to 300 mg | 0.18 |
| 450 mg | 0.37 |
| 600 mg | 0.37 |
| **Rifapentine** |  |
| 150 to 600 mg | 0.65 |
| 750 to 900 mg | 0.97 |

**Table H: Prevalence of positive TST stratified by country and age group, used to estimated number with TB infection (TBI) among HHC randomized to Strategy 3 (No-TST)**

| **Country,**  **Age group** | **TST results: Strategy 1&2 combined** | | | **Estimated TBI: Strategy 3** | |
| --- | --- | --- | --- | --- | --- |
|  | **HHC in group (N)** | **With Pos TST (N)** | **Percent Pos TST (%)** | **HHC Total (N)** | **HHC with Positive TST** |
| **Benin** |  |  |  |  |  |
| 5-10 | 214 | 63 | 29% | 112 | 33 |
| 11-17 | 232 | 75 | 32% | 125 | 40 |
| 18-24 | 122 | 43 | 35% | 57 | 20 |
| 25 and older | 252 | 110 | 44% | 125 | 55 |
| TOTAL (Benin) | 820 | 291 |  | 419 | 148 |
|  |  |  |  |  |  |
| **Brazil** |  |  |  |  |  |
| 5-10 | 33 | 19 | 58% | 26 | 15 |
| 11-17 | 45 | 31 | 69% | 25 | 17 |
| 18-24 | 26 | 17 | 65% | 11 | 7 |
| 25 and older | 71 | 41 | 58% | 51 | 29 |
| TOTAL (Brazil) | 175 | 108 |  | 113 | 69 |
| Abbreviations: HHC: Household contact; TST: Tuberculin skin test; TBI: TB Infection (in this study assumed to be a positive TST)  Notes: Criteria for positive TST, In Benin: Induration of 10+mm, and in Brazil: Induration of 5+mm. | | | | | |

**References for Supplemental tables**

1. Alsdurf H, Oxlade O, Adjobimey M, Ahmad Khan F, Bastos M, Bedingfield N, et al. Resource implications of the latent tuberculosis cascade of care: a time and motion study in five countries. BMC Health Services Research. 2020;20(1):341.

2. Adjobimey M, Ade S, Wachinou P, Esse M, Yaha L, Bekou W, et al. Prevalence, acceptability, and cost of routine screening for pulmonary tuberculosis among pregnant women in Cotonou, Benin. PLOS ONE. 2022;17(2):e0264206.

3. Jabour E, Pinto M, Steffen RE, Dockhorn F, Pelissari DM, De Souza NM, et al. Estimated costs of tuberculosis-related activities and interventions in Brazil, 2023. 2024. In press.

4. World Health Organization. WHO-CHOICE Tools. Accessed at <https://www.who.int/teams/health-financing-and-economics/economic-analysis/costing-and-technical-efficiency/quantities-and-unit-prices-(cost-inputs>).

5. Oxlade O, Benedetti A, Adjobimey M, Alsdurf H, Anagonou S, Cook VJ, et al. Effectiveness and cost-effectiveness of a health systems intervention for latent tuberculosis infection management (ACT4): a cluster-randomised trial. Lancet Public Health. 2021;6(5):e272-e82.

6. Steffen RE, Caetano R, Pinto M, Chaves D, Ferrari R, Bastos M, et al. Cost-effectiveness of Quantiferon®-TB Gold-in-Tube versus tuberculin skin testing for contact screening and treatment of latent tuberculosis infection in Brazil. PLoS One. 2013;8(4):e59546.

7. Bastos ML, Campbell JR, Oxlade O, Adjobimey M, Trajman A, Ruslami R, et al. Health System Costs of Treating Latent Tuberculosis Infection With Four Months of Rifampin Versus Nine Months of Isoniazid in Different Settings. Ann Intern Med. 2020;173(3):169-78.

8. Brazilian National Health Surveillance Agency. Medication index price list. Avaiable at <https://www.gov.br/anvisa/pt-br/assuntos/medicamentos/cmed/precos>.
