# Supplementary material for: Rapid molecular testing or chest X-ray or tuberculin skin testing for household contact assessment of tuberculosis infection: A cluster-randomized trial
Source: PLoS Med. 2025 Jul 28;22(7):e1004666. doi: 10.1371/journal.pmed.1004666 (PMC12316388; doi:10.1371/journal.pmed.1004666)
Supplement: S1 Protocol — (DOCX) [file pmed.1004666.s004.docx]

**GXT –** **GeneXpert or chest-X-ray or Tuberculin skin testing for household contact assessment:**

**a cluster randomized trial**

**Main objective**:

To compare across three different strategies for contact investigation the proportion of household (HH) contacts treated for active and latent tuberculosis (TB), time to treatment initiation, adverse events, as well as costs from the perspective of the health system and patient.

**Summary of the 3 strategies compared:**

*Children under 5 years old and people living with HIV (known or detected through study procedures) will be excluded from this study as they should receive an expedited strategy as recommended by WHO. Adults older than 50 years old will also be excluded; they will be investigated and treated for LTBI on an individualized basis by their provider after judging the balance of risks and benefits.*

Study participants will be randomized to:

*1. Standard care (control arm)*: Participants will receive symptom screening and tuberculin skin testing (TST). If symptom screen positive and/or TST positive, they undergo chest x-rays (CXR). If CXR abnormal, they undergo microbiological investigation. If CXR normal or if microbiological investigation negative, TST positive receive latent TB infection (LTBI) treatment. If microbiological investigation is positive they will be offered treatment for active TB.

*2. Rapid Molecular Test (RMT – all site used GenXpert MTB/RIF – or GX)*: Participants follow an algorithm similar to the standard care, however participants with positive symptom screen and/or positive TST will receive GX (i.e., GX replaces CXR in standard care algorithm). GX positive are considered to have active TB. TST positive and GX negative receive LTBI treatment. If an individual is not able to provide sputum, they will undergo a CXR.

*3. CXR for all/NoTST*: Participants will receive symptom screening and CXR. No TST will be performed. If CXR abnormal or symptom positive, they undergo microbiological investigation. If the CXR is normal, and/or microbiological investigations negative – they receive LTBI treatment as per national guidelines. If microbiological investigation is positive they will be offered treatment for active TB.

**Specific objectives**:

To compare outcomes that result from three different strategies for the management of HIV uninfected persons aged 5-50 years who are HH contacts of newly diagnosed microbiologically confirmed active pulmonary TB.

Primary Outcome:

Among HH contacts of patients with newly diagnosed active pulmonary TB (index TB patient), who are eligible (measured or estimated) for LTBI therapy, the proportion starting LTBI therapy within 3 months of the index TB patient starting active TB treatment.

Rationale: The primary endpoint will be the number of HH contacts who start latent TB therapy within 3 months of the date of start of active TB treatment in the index TB patient (the member of their household who was diagnosed with active TB and because of whom the contact investigation was performed). Because household contacts are at risk to progress to active TB within months after exposure and infection, all TB programs recommend that contact investigation is completed, and contacts started on latent TB therapy rapidly - as soon as possible once active TB has been excluded. For this study we will use a benchmark that contacts should begin LTBI therapy within 3 months of the date of start of treatment of the Index HH member who has active TB. The date when the index case TB patient is started on active TB treatment is easy to identify and standardized, whereas the date of diagnosis of active TB can be difficult to establish as it depends on the modality of diagnosis.

Secondary outcomes:

1. Societal costs (health system and patient costs) of the full cascade of care - from initial identification to LTBI therapy completion.

2. Prevalence of microbiologically confirmed and clinically diagnosed active TB – detected as part of the initial contact investigation, who initiate treatment within 3 months of the date of randomization of the contact.

3. Prevalence of positive TST (>5 mm or >10 mm) – overall, and by age group.

4. Incidence of grade 1-4 adverse events related to LTBI therapy.

5. Completion of LTBI therapy – defined as having taken at least 80% of doses in 120% of allowed time.

6. Sensitivity and specificity of CXR reading by usual providers in each study site (reference standard will be readings by an external review panel).

7. Prevalence of active TB diagnosed using CXR in participants who cannot produce a sputum sample.

**Background and rationale for the study:**

**Impact of latent TB infection**

There are several unique features of the pathogenesis of TB that make global eradication of this disease very challenging. TB is transmitted through the airborne route from a person with active and contagious pulmonary TB to an uninfected person in close proximity. If infection is acquired during this exposure, then in more than 95% of persons, disease does not develop right away. Instead the newly infected person will develop latent or dormant infection, which can reactivate to cause active disease months to decades later. It has been estimated that one quarter of the world’s population has latent or dormant tuberculosis [1]. This acts as a huge reservoir from which active symptomatic and contagious TB develops. Active TB that is untreated is fatal in a high proportion of individuals, although if diagnosed and treated, mortality is much lower. However, substantial morbidity can occur despite treatment: several studies in low and middle-income countries (LMIC) demonstrated that persons who recovered from active TB had an average loss of lung function of 25% – equivalent to the effects of heavy cigarette smoking for more than 25 years. Hence, prevention of TB, through treatment of LTBI, could provide very important potential individual benefits – by preventing this morbidity and mortality. TB prevention could also provide important public health benefits. In a modelling study, officials at the World Health Organization (WHO) estimated that treatment of LTBI was essential to achieve long-term reductions in global TB incidence [2]. In view of the individual and public health benefits, a recent United Nations high level conference on TB set a target that more than 30 million individuals should be treated for LTBI over the next 5 years. This ambitious goal will require massive scaling up of LTBI diagnosis and treatment – which will be challenging, if not impossible, with current approaches to diagnosis and treatment of latent TB.

**Diagnosis of latent TB infection (LTBI)**

The diagnosis of LTBI relies upon detection of an immune response to antigens from *M. tuberculosis*, the microorganism that causes TB. Two tests are available to detect antigens: the Tuberculin Skin Test (TST), first introduced in 1908, and the interferon gamma release assays (IGRA), which have been in clinical use for the past 15-18 years. The IGRA’s measure the immune reaction to more specific TB antigens and are *ex-vivo* tests. Despite its relative antiquity, the TST remains the most commonly used test for LTBI diagnosis in the world. The advantages of the TST include its low cost and feasibility in resource-poor settings because there is no need for complex laboratory equipment or procedures, nor for highly trained technicians, although trained health care personnel and a functioning cold chain are necessary. One disadvantage is that the TST is less specific, but two meta-analyses have summarized that TST can be used to predict benefit of LTBI therapy [3, 4]. Although there are some inconsistencies, the majority of the published studies have demonstrated that in those who are TST positive, LTBI therapy is significantly beneficial, but is not beneficial when given to those who are TST negative.

Despite this, the current WHO recommendations allow TB programs in resource-poor settings to give LTBI therapy to persons living with HIV, and children under five years – even in the absence of TST [5]. This reflects the high risk of disease in these two populations if LTBI treatment is not given, and the operational challenges of performing the TST in LMIC. These include the difficulties of training and quality assurance of TST administration and reading, and a global shortage of tuberculin material. This last problem was due in part to production problems of a major supplier – *Statens Serum Institut* (SSI) in Copenhagen. The production problems are now resolved, but the global shortage remains, and because of this, the price of tuberculin test material (PPD) has risen considerably. The problems of quality assurance of TST are well recognized – although considered a reliable test ‘in good hands’ – those good hands require training and ongoing supervision – as with any diagnostic test. But, the most important limitation is that only about 10% of those with a positive TST will ever develop active disease. Hence, ten persons with presumed LTBI must be treated to prevent one from developing disease.

The IGRA’s have become increasingly popular over the past 15 years – due in large part to their improved specificity – a major advantage in high income, low prevalence settings. However, the unit costs for these assays are higher than for the TST and are less accessible than TST in most resource-limited settings due to the need for complex laboratory equipment and well-trained technicians. In addition, the IGRA’s are equally inefficient as the TST for predicting who will develop disease. Several meta-analyses and large prospective studies have shown the IGRAs to have little or no difference in their ability to predict future active TB [6-9]. Current WHO recommendations state that IGRA or TST may be used, although several potential advantages of TST for resource-limited settings are listed in the same document [5].

**Treatment of LTBI**

The inefficiency of LTBI diagnosis would be less important if treatment of latent TB was quick, inexpensive and safe. However, for the vast majority of persons treated for LTBI in LMIC, current treatment has none of these characteristics. The current standard treatment, recommended by the WHO, is 6 months Isoniazid (6H). This is obviously long and also can lead to serious side effects including potentially fatal liver toxicity, particularly in older adults. Even in high income settings with close monitoring and follow-up, liver failure (requiring liver transplantation) and deaths still occur [10, 11]. In addition to these problems (or perhaps because of them), less than 50% of persons complete 6H or 9H as prescribed, under routine conditions in many programs [12].

These problems with INH have motivated considerable work in the past 20 years to develop and test new, shorter regimens. Most of these regimens have included rifampin or rifapentine – which have greater bactericidal activity, allowing shortening of active TB treatment. Three rifamycin-based regimens have been shown, in randomized trials, to have at least as good efficacy for TB prevention as 6 or 9 months INH. The regimen of 3-4 months INH and rifampin (3HR) has the same efficacy and adverse event profile as 6 months INH [13]. A regimen of 3 months INH and rifapentine (3HP) given once weekly for 12 doses had non-inferior efficacy compared to 9H, and similar safety in large scale randomized trials [14]. In these trials, the overall rate of grade 3-4 adverse events, or adverse events requiring regimen change, were more frequent with 3HP than with 9H [14, 15], but liver toxicity was significantly less with 3HP [14]. The third alternative is 4 months daily rifampin. In recent trials, when compared to 9H, this regimen has been shown to have non-inferior efficacy for TB prevention in largely HIV uninfected adults [16] and children [17]. Very importantly, this is the only LTBI regimen that has consistently shown significantly lower rates of grade 3-4 adverse events (or drugs stopped due to adverse events) when compared to the standard 9H [16-18]. On the basis of these trials, and associated meta-analyses, in LMIC WHO has recommended use of 6INH or either 3HR or 3HP, and country National TB Programs (NTP’s) have started adopting them. WHO also recommended these regimens, as well as 9H, and 4R in high income countries [5].

**Importance of household contacts**

HH contacts are well recognized to be at high risk for active TB. This includes prevalent active TB – detected at the time of initial contact investigation – and incident active TB that occurs within the next 2 to 5 years. In systematic reviews, among HH contacts in LMIC, the prevalence of active TB ranges from 3% to 5% [19, 20]. For this reason, screening HH contacts for active TB is considered a high priority for virtually all TB control programs in all LMIC as this is a very high yield active case finding strategy. Treatment of LTBI among these HH contacts may also have an important potential benefit as the prevalence of LTBI exceeds 50% in LMIC [19, 20]. Hence, identification and investigation of contacts is a high-yield activity, for detection and treatment of active as well as LTBI.

The investigation and treatment of all the HH contacts has other important advantages – this approach can provide important health and financial benefits to the entire family. The occurrence of TB can have very serious effects on the family including the well described catastrophic costs related to loss of income and costs for care. [21, 22]. By investigating and detecting both active and latent TB – further catastrophic costs will be avoided, and the amplified health consequences due to occurrence of TB in other HH members prevented. Hence this approach is in line with a more holistic ‘family health’ approach. In addition, this approach is much more efficient – TB care can be provided to all HH members at once, during the period of time the index TB patient is still receiving care – maximizing the impact and cost-effectiveness of health system interventions, such as home visits, or minimizing patient costs due to travel for follow-up visits.

**The cascade of care in LTBI – Methods and problems of contact Investigation**

The diagnosis and treatment of LTBI – in HH contacts or others with LTBI - represents a complex cascade of care. In a recent systematic review, we demonstrated that substantial losses and dropouts occur throughout that cascade [12], substantially reducing the potential benefit of LTBI management. Of all those with LTBI in the studies included in the review, only 18% completed investigation and therapy appropriately.

Two aspects of management of LTBI – need for CXR and need for TST - are controversial, as both can create important barriers to LTBI initiation, thereby reducing the individual as well as public health benefits. WHO updated its guidelines for LTBI management in 2018 [5]; this included detailed recommendations for investigation to exclude active TB which is important to avoid inadvertent mono-therapy of patients with undiagnosed active TB, as this can lead to the emergence of resistance. The algorithm suggested in HIV negative persons aged 5 and older is based on extensive experience in randomized trials. In these trials, patients underwent screening to exclude active TB including symptom assessment and CXR. Although not necessarily 100% sensitive to detect all prevalent active TB, ***this testing was sufficient to prevent the development of resistance in those who received INH treatment*** [23, 24]. This is the fundamental objective – if a screening algorithm is sufficient to identify (and therefore exclude) those who will develop resistance if given INH mono-therapy, there is no justification for further testing. Symptom screen has acceptable sensitivity to detect prevalent active TB among HIV infected individuals who are not treated with anti-retroviral therapy (ART) [25] and child contacts aged less than 5 years old [5]. However, symptom screen alone is less than 50% sensitive to detect active TB in adults and children aged 5 and older who are HIV uninfected, or HIV infected person on ART [5, 26]. In these same populations, CXR screening has sensitivity as high as 98%, if any abnormality triggers further investigation. This may explain why the current algorithm is sufficient to avoid development of INH resistance. However, CXR services are not accessible in many settings and even if accessible, the cost for a CXR often falls on patients [27] and is often prohibitively expensive. For example, in the proposed study sites in Benin, the NTP and government health services do not cover the full costs for CXR; therefore, patients must bear all or part of the cost. In Benin patients pay the equivalent of 10 Euros for one CXR. In a family with 6 HH contacts, the cost of CXR for all contacts will exceed the average one-month salary for an unskilled worker. This is clearly not affordable, particularly as part of a preventive strategy. Hence the requirement of a CXR as part of the pre- LTBI treatment algorithm results in a major barrier preventing HH contacts from starting on potentially beneficial LTBI treatment.

An alternate strategy is to use GeneXpert equipment, with the Xpert MTB/Rif test (hereafter labelled GX) to replace CXR to exclude prevalent active TB. This will make the algorithm less complex (fewer steps), and may increase the number of patients who successfully complete all steps in the algorithm. GX, a recent addition to the tuberculosis diagnostic armamentarium, is a cartridge-based test that utilizes nucleic acid amplification techniques (nested PCR) to detect *M. tuberculosis* DNA in sputum samples within 2 hours. This test also can detect rifampin resistance if tuberculosis is present. The availability of this test has steadily increased over the last 10 years in LMIC. Sensitivity is better than that of direct microscopic examination of a sputum for acid fast bacilli (AFB smear), although less than TB liquid culture. GX is also less sensitive, but more specific than CXR, and the result is much more reproducible. Since the GX cost is always covered by TB programs, from a patient perspective, this is more attractive than CXR. In addition, the high sensitivity and 2-hour turnaround time means that this can be used as a rapid rule out test in HH contacts.

Disadvantages to this strategy include the lower sensitivity of GX in patients with minimal active disease (so called pauci-bacillary disease), raising concerns about giving therapy for latent TB infection to patients with negative GX but minimal active disease. However, based on earlier studies of latent TB therapy there appears to be a considerable margin of safety. The first United States public health study conducted in 1958-1962, randomized children with parenchymal infiltrates and hilar lymphadenopathy to placebo or INH monotherapy. Children with these findings on CXR nowadays would be considered to have active disease. Despite this evidence of significant active TB, children treated with INH alone had very high success rates and no reported emergence of resistance [28]. Another group would be patients with symptoms who are unable to produce sputum. This could raise concerns about missing minimal active TB, so a CXR will be done for all patients with TB symptoms who cannot produce a sputum sample. another disadvantage is false positives since prevalence is low.

**Prior relevant research by the team**

Completion, safety and efficacy of 4 months rifampin (4R) vs 9 months INH (9H): Between 2001 and 2018, we conducted a series of three clinical trials to compare treatment completion, serious adverse events and efficacy in TB prevention of 4R and 9H. The phase 2 and phase 3 studies (completed in 2007 and 2018 respectively) were conducted in Brazil and Benin among other sites. In these studies, we demonstrated that in these 2 countries, patients’ acceptance of LTBI diagnosis and treatment was high, their tolerance and completion of both treatments were good, although completion and safety were significantly better with 4R. These findings suggested that latent TB management was well accepted by providers and patients in these settings. The results of these trials were fundamental for the current WHO recommendations.

A cluster randomized trial of a public health intervention to improve the Cascade of care in LTBI (ACT4): During the conduct of the 4Rvs9H trial, a number of major challenges in the LTBI cascade of care were identified. These motivated ACT4 – a cluster randomized trial of a public health intervention to improve the latent TB cascade of care at health facilities in Benin, Ghana and Brazil, as well as in Canada, Indonesia and Vietnam. Initial cascade of care analyses in Benin and Brazil revealed that almost no HH contacts were initiating LTBI treatment (let alone completing it). The ACT4 study revealed different problems at each study site; as part of the study, local solutions were identified and implemented by stakeholders at each site. These were successful in resolving barriers to LTBI diagnosis and treatment, and when these potential barriers were identified and removed, we found that HH contacts in these settings were enthusiastically adherent to the various steps in the cascade of care in latent TB, resulting in substantially greater uptake of LTBI therapy in all settings.

However, at most sites the performance of TST and access to CXR prior to initiating LTBI therapy were problems that were very difficult to resolve. The TST was not performed in most settings – in part based on the recommendations of WHO, and in part because of cost and complexity (and also a global shortage of tuberculin testing material). Hence staff at the sites had to be trained and a quality assurance program initiated to maintain high quality performance of TST. The CXR was also an important additional barrier, because even if available, CXR was not paid by the TB programs, so costs had to be borne by TB patients or their contacts. For the ACT4 project, CXR were paid by research funds, but this is clearly not sustainable. In other settings, a waiting list for CXR delayed substantially LTBI treatment initiation. Therefore, either the CXR has to be abandoned as an integral part of LTBI investigation or WHO must adopt a policy that CXR are an essential service to be provided for TB patients and their contacts.

**Summary of evidence**

In summary, current WHO recommendations allow TB programs in resource-poor settings to manage latent TB without testing in under five child contacts and HIV infected of all ages. This is because the guidelines group considered that the risks from not receiving LTBI treatment in those with latent TB outweighed the risks of unnecessary therapy in those without LTBI treatment. We believe the same argument can be applied to all contacts under 50 years – as this group is also at high risk to develop disease. Current policy and practice in many LMIC is that HIV uninfected HH contacts aged 5 years and older do not receive LTBI treatment. This is because incorporation of TST and CXR into the test and treat algorithm for this group has had the unintended effect of reducing the likelihood that HH contacts with LTBI are treated at all. In a nutshell, the current policy has deprived a high-risk group of significant potential benefits, because of concerns about potential harms from overtreatment or misdiagnosis (i.e. treating for LTBI when it is in fact active TB). From our prior research studies in these settings, we know that LTBI treatment is well accepted with high rates of completion among HH contacts. However, the difficulties of access to affordable CXR and high quality reliable TST in these settings are major impediments to long term success of our previous findings. We believe the balance of benefits and risks will favor LTBI treatment of HH contacts in the absence of TST, and also that GX may be a very satisfactory replacement for CXR. However, the advantages and disadvantages of the different approaches we propose to test in this study may vary depending on the resources available at each setting, as well as the prevalence of LTBI and age structure in the populations of HH contacts.

**STUDY DESIGN, INTERVENTIONS AND RATIONALE**

This will be a cluster randomized trial with three arms of equal size; clusters will be defined as all the household contacts of patients with newly diagnosed active pulmonary TB. The first eligible member of the HH who provides signed informed consent to participate will be randomized to one of the three strategies. All subsequently enrolled members of the same HH will be assigned to the same arm. In all strategies, if the treating team believes that there is sufficient clinical, radiologic and microbiologic evidence for the diagnosis of active TB, the HH contact will receive treatment for active TB. If other respiratory diseases (ie. pneumonia, asthma etc) are suspected, then these will be investigated and treated by the primary provider.

*As mentioned above, HH contacts who are <5 years old, and/or HIV infected should complete expedited investigations as recommended by WHO and so will be excluded from the study. All potential study participants will be asked about known HIV infection, as well as current therapy with ART. As well contacts may be tested for HIV infection – those found to be positive will be excluded – including post randomization. Other members of the HH aged 5-50 years will receive LTBI treatment as per national algorithms. HIV uninfected HH contacts aged over 50 years will also be excluded, as LTBI treatment would not be given routinely to them – to enhance safety. On an individualized basis the provider may recommend therapy if they judge that the risks of disease outweigh risks of adverse events.*

**Rationale for cluster study design**

A family-based approach to TB control is proposed, as we see this as the most feasible next step in TB endemic areas to truly begin to prevent future cases of active disease. This has the advantage of being a highly feasible strategy, for active case finding, and LTBI detection and treatment compared to population level testing. This is because the TB programmes in most countries, and certainly in the participating countries, already identify and screen HH contacts of newly diagnosed patients with active TB – hence the proposed activities are an extension of those activities, rather than entirely new ones.

***Strategy ONE: Standard:*** *This strategy is based on the current WHO recommended algorithm for HH contacts that are HIV negatives and age > 5 years.* *TST and microbiologic testing are generally paid by the TB program, but in Benin, in normal practice, the HH contacts pay for CXR. For study participants, these CXR costs will be paid by research funds.*

Symptom screen and TST at the time of the first interview. (An IGRA would be an acceptable alternative, but in the 2 countries where this study will be conducted, IGRA testing is not accessible in the public health system).

If *no symptoms*: participants with negative TST will be discharged. The cut-point for negative/positive TST will be according to NTP policy in each country. If positive TST, then CXR will be done.

If *symptom screen is positive* (cough, sputum, fever, anorexia/weight loss), participants will undergo a CXR. (Note: In all situations in Strategies 1 & 3, when a CXR is indicated for a female HH contact of child bearing age, she will be questioned regarding possible pregnancy, and if confirmed (or possible) will undergo CXR with appropriate shielding). If the CXR is abnormal, the HH contact will have microbiologic investigations. These microbiological investigations will comply with NTP policy and procedures in each country. In Benin, when pulmonary tuberculosis is suspected, two sputum samples are taken – the first sample is taken immediately on the spot, and the second is collected by the patient at home the next day upon waking. Refer to appendix 1 for details of procedures in Brazil, where this will also be standardized for all study participants. If the CXR is normal, and symptoms have resolved after a week – then TST negative will be discharge and TST positive will be recommended to start LTBI therapy. If the CXR is normal but TB symptoms persist, then microbiological testing will be done. If the microbiological tests are negative for active TB, then participants will be discharged if TST negative, or recommended to start LTBI therapy (if TST positive).

IF TST (or IGRA) indicated, (Note: for Strategy 1&2 persons with history of prior treatment for active TB will not have TST, but will have a CXR to exclude active TB).

*TST negative* - will have no further investigation. (*Brazilian guidelines recommend a second TST 8 weeks later. This will not be mandated by the study protocol – to be consistent in all countries and with WHO guidelines. However, if providers wish to do this second TST – this can be done and therapy given if the second TST is positive).*

*TST positive* - will have a CXR (if not done already). If CXR abnormal they will have microbiological testing as above. If CXR normal, and no history of prior TB treatment, they will be recommended to start LTBI therapy.

For Strategy 1 and 2 – the use of the TST may be new in some clinics and for some HCWs. Training will be done for TST administration and reading, during initial site training. The “mTST tool” will be used as part of the quality assurance for the TST – at all sites (see link for video on mTST instructions <https://www.youtube.com/watch?v=xmCRJ44TmpQ>, and appendix 2 for instruction manuals for mTST).

**Figure 1: SCHEMATIC OF STRATEGY 1: Standard** *(based on Algorithm in WHO LTBI guidelines 2018)*


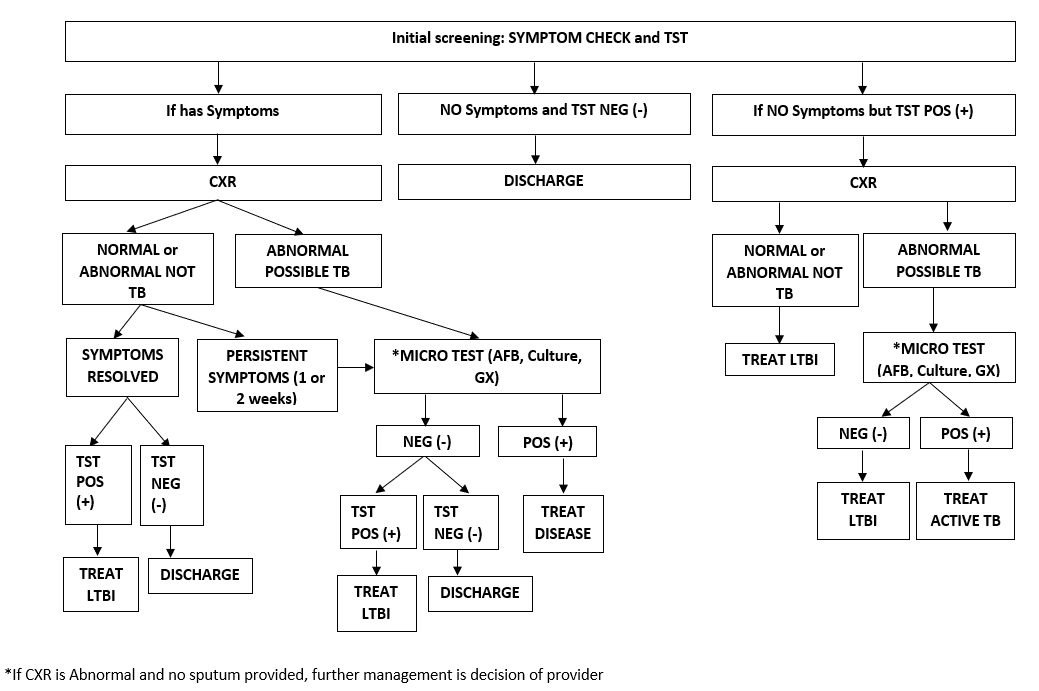


***STRATEGY TWO: RMT & TST Strategy****:*

*The main difference from Strategy 1 above is that a rapid molecular test (GX) replaces CXR everywhere that CXR would normally be done. TB program pays for all TST and GX.*

Symptom screen at the time of the first interview.

No symptoms will have TST (or IGRA, but as described above this will be TST in study sites)

Positive symptom screen (cough, sputum, fever, anorexia/weight loss) will undergo a GX.

GX positive – treat active TB

GX negative - TST (or IGRA)

IF TST (or IGRA):

TST negative - will have nothing further.

TST positive - will have a GX (if not done already).

GX positive – will have a chest-x-ray as part of an assessment of the diagnosis and need for treatment of active TB. Treatment will be at the discretion of treating clinician/team (see below – for rationale).

GX negative - will be recommended to start LTBI therapy.

It is predicted that even after careful training, approximately 40% of adolescents and adults would not be able to produce a spot sample of sputum and an even higher proportion of children aged 5 to 10 years will be unable to produce a sample sputum. In these participants this may result in missed active TB with the GX strategy. In the Vietnamese ACT3 population-based study, sensitivity of GX to detect pulmonary active TB among household contacts was only 40%, compared to a reference standard of 2 sputum samples sent for liquid TB culture. Hence, there is a real risk that active TB can be missed with the GX strategy, although an argument could be made that this will likely be minimal active TB. We think it better to ascertain this through a secondary procedure with appropriate secondary analysis. Participants who are randomized to the GX strategy and are unable to produce a sputum sample - after standard efforts, will have a CXR. If the CXR is abnormal and judged possibly or probably active TB such that microbiologic investigations are necessary, these will be done following procedures outlined for the other two arms. In a planned secondary analysis, we will examine the proportion of participants in the GX arm who are unable to produce sputum and: (i) are judged to have possible or probable active TB on CXR; or, (ii) are treated for active TB ; or, (iii) have positive sputum cultures.

Similarly, CXR and other investigations can be performed at the discretion of the treating physician/team if other respiratory diagnoses are suspected. Or, if CXR abnormal or CXR normal plus symptoms, but microbiologic investigations are negative, then medical evaluation can be done to exclude other respiratory illnesses. (Indeed, in all three strategies, the investigations mandated by the protocol are the minimum required. But the treating team can, at any time, order additional investigations and prescribe non-TB related treatment, if they feel these are clinically warranted). All non-protocol mandated investigations and treatment will be recorded, and the costs added in the calculation of total costs for each strategy, regardless of whether these added costs are borne by the public health system or the patient.

**Figure 2: SCHEMATIC OF STRATEGY 2: RMT replaces CXR**
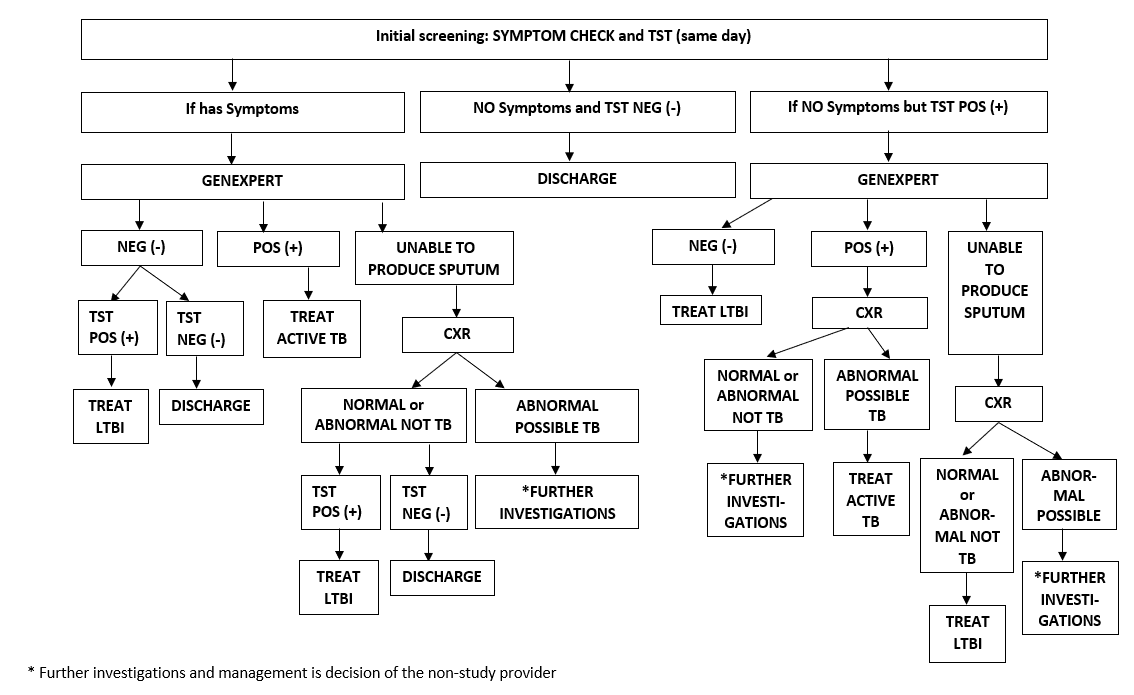


Rationale for RMT intervention:

The unit cost of GX is similar to that for CXR, but in all countries the cost for this test is borne by the TB program – an important advantage to patients. This strategy may prove to be more expensive for the TB program/health system, but will also be more rapid and efficient in detecting active disease as this skips the step of CXR which is then followed by microbiologic investigations. From the TB program’s perspective, GX offers the advantage of good sensitivity (better than AFB smear). For this study, GX is already available at proposed sites in Benin and Brazil. If participants have symptoms but are unable to produce sputum it is plausible that these procedures may miss some cases. However, studies have demonstrated that training of health care staff about how to obtain sputum from patients will enhance yield [29]. (refer to appendix 3 for example patient instructions from Zimbabwe; this will be adapted for use in the two countries). If staff have been trained in instructing patients, and no sputum is produced, then active TB is unlikely in asymptomatic patients; however, we will routinely obtain a CXR in all these HHC (see above). In addition, providers will be encouraged to exercise their clinical judgement in care of symptomatic HH contacts. Therefore, they may decide to treat such patients with antibiotics or other therapy – based on their clinical judgement. This would be in accordance with current WHO recommendations for patients with “TB symptoms” but negative microbiologic investigations. If, after 1-2 weeks, the symptoms have resolved then LTBI therapy can be started. If the HH contact is still symptomatic, then another attempt will be made to obtain a sputum sample. If no sputum sample is obtained, then providers may decide to order other investigations such as a CXR, or other treatment.

On the other hand, if the GX is positive, we must consider possible false positive results as GX has a specificity of 98%. If the contact has a positive GX and TB symptoms – the GX will be considered true positive, given the higher pre-test likelihood disease. However, if the contact has no symptoms and a positive GX they will also have a CXR routinely to assist in the clinical decision making. This is because GX has a 2% false positive rate. In asymptomatic HH contacts – the expected prevalence of active TB is low – estimated to be less than 2%. In these persons the positive predictive value might be less than 50%. Hence a CXR will be mandated – to assist in the clinical assessment of these contacts, and reduce the risk that contacts with a falsely positive GX receive unnecessary treatment for active TB.

***STRATEGY 3: CXR ALL, no TST***:

This group will undergo CXR regardless of presence of symptoms. If the CXR is abnormal, or CXR normal and symptoms then microbiologic investigations will be done. These investigations will be as per national TB policy and procedures, as above. If CXR abnormal or CXR normal plus symptoms, but microbiologic investigations are negative, then medical evaluation will be done to exclude other respiratory illnesses. If no sputum is obtained for microbiologic investigations, then usually this will be considered a negative microbiologic result. However, this will be up to the treating team/provider – if they agree – then LTBI therapy can be started. But if they feel that additional investigations are warranted, then they may follow the current WHO algorithm for TB suspects – which is to treat for an alternative diagnosis (usually oral antibiotics for 7-10 days for a respiratory tract infection) with repeat evaluation after 14 days. If persistent symptoms, then repeat sputum examinations are recommended. If at any time TB is confirmed or considered likely by the treating team/provider, then active TB treatment is started. Once active TB is considered excluded by the treating team, then the HH contact will be recommended to start LTBI therapy. All *microbiologic tests are paid by the TB program, but in Benin, in normal practice the HHC pays CXR cost. Study participants: CXR will be paid by research funds.*

**Figure 3: SCHEMATIC OF STRATEGY 3 (CXR all, no TST)**


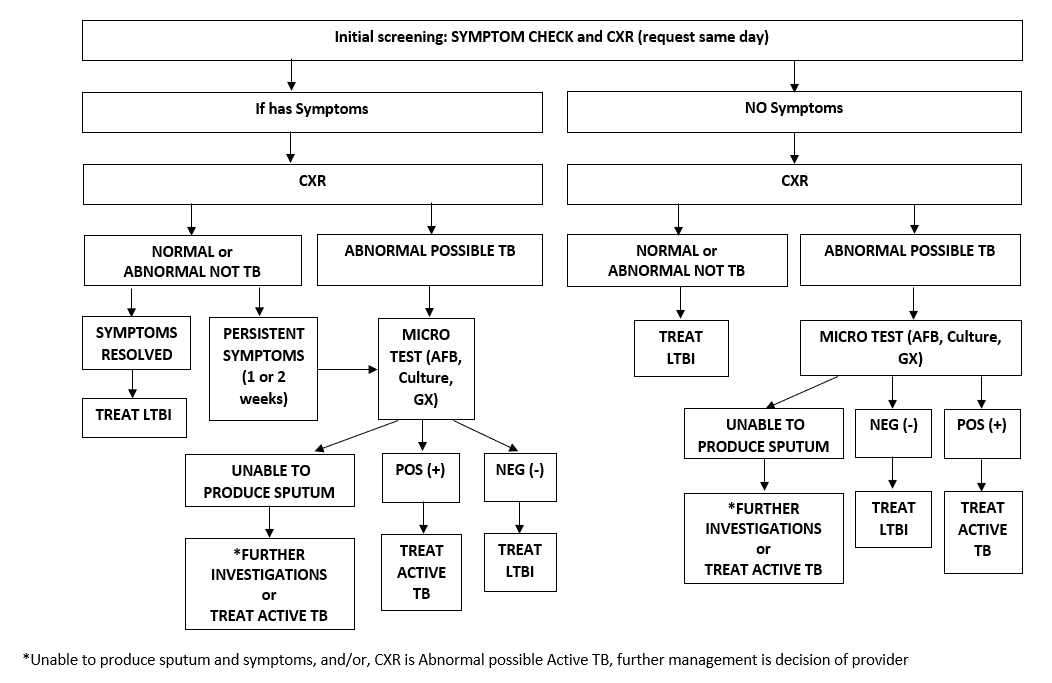


Rationale for CXR all/noTST intervention:

In this strategy CXR will be performed for all HH contacts – so persons with prevalent active TB will not be missed. However, the overall program costs will be higher, due to costs related to the CXR. For ethical reasons the study must bear costs of CXR for participants, however if this arm were shown to be best strategy the cost implications (i.e. need for publicly funded CXR) would need to be addressed. Since the CXR has long been discouraged by WHO policy this study findings would be presented to WHO, and the National TB programme managers in the participating countries, as part of the knowledge translation efforts.

The other disadvantage of this strategy is that HH contacts **without** LTBI are treated – which results in unnecessary costs (patient and health system) and risks to patients from adverse events without potential benefits. However, this approach warrants consideration for several reasons: 1) TST may be inaccurate due to poor technique – due to lack of trained personnel, and/or lack of a quality assurance program; 2) Stock-outs of tuberculin test material due to recurrent global shortages make TST frequently unavailable; 3) Delays and dropouts plus added costs are associated with TST, due to need for multiple patient visits for TST administration and reading. Without need for a TST, LTBI therapy can be started the same day as symptom screen and CXR (one stop shop); 4) Among the highest risk groups of child contacts aged <5 years, and HIV infected persons the WHO recommends TST, but makes it clear that if TST cannot be performed, for any of the reasons listed above, that LTBI therapy should be given – since the benefits of therapy outweigh the risks in these groups. It may be argued that the same is true for all HH contacts.

A further consideration is the expected prevalence of positive TST among HH contacts; as prevalence increases the no-TST strategy becomes more advantageous. One systematic review estimated the pooled prevalence of LTBI among HH contacts in LMIC to be 51% [19, 20]. However, data from previous work at our study sites shows that prevalence of positive TST among HH contacts of all ages is 51% in Brazil (M Bastos unpublished), and 72% in Benin (M Adjobimey unpublished). This means that the number unnecessarily treated would be less in these high TB incidence/ high LTBI prevalence study settings. In addition, the HH contacts most likely to have a negative TST are the youngest. This is the group who are also the least likely to have any serious side effects with any of the LTBI regimens [10, 30, 31]. Hence, the risk of unnecessary side effects from treating LTBI in persons who do not have LTBI should be low.

Another way that we have reduced the risk to participants is that we have limited routine provision of LTBI treatment to HH contacts aged younger than 50 years, as the rates of adverse events is higher in those aged over 50 years [30, 31] although providers can recommend therapy if they feel the benefits outweigh the risks on an individual basis. A final mechanism to reduce risks as well as time and cost burden on study participants will be to introduce shorter regimens such as 4R, 3HP or 3HR instead of 6H as standard LTBI treatment. In this way, all HHC treated within this study would receive the same shorter regimen –i.e. the regimen would not be randomized. This has been accepted by the National TB Program in each country, as this will provide pilot programmatic experience to demonstrate the advantages – to patient and programs - of use of shorter rifamycin based LTBI therapy.

In Benin, 3HR will be used – as the fixed dose combination formulations for adults and children are available in all health centres. As reviewed earlier, 3HR has better completion rates than 6INH, which should result in better effectiveness, and lower program as well as patient costs, since fewer follow-up visits are necessary. This would help compensate for the increased work load of more contacts being treated. An added advantage of 3HR is that it’s easy to implement in all settings as the formulations – for adults and children are already available in all settings, and staff are familiar with dosing and administration, as they are used for treatment of patients of all ages with active TB. In Brazil, 4R may be used – as this is approved for use by the National Programme. Of all the currently recommended short regimens tested in randomized trials, only 4R has had significantly lower overall rate of all serious adverse events, or of hepato-toxicity than 9H or 6H. Hence use of 4R should reduce time and cost burden on patients and staff, plus reduce risk of AE.

**STUDY POPULATION**

**Study site (clinic) selection**

We will use a checklist (refer to appendix 4) for each health facility that will focus on the number of index TB patients diagnosed at the facilities, and their capacity to conduct all the steps of the LTBI cascade of care. These include contact investigation, Tuberculin skin testing and reading, performance and interpretation of CXR, performance of GeneXpert and of subsequent microbiologic testing, LTBI treatment initiation and follow-up. For a clinic to be selected, they do not necessarily need to have CXR or GeneXpert facilities on site, but they must make arrangements that CXR and/or GeneXpert can be performed on household contacts in a nearby health facility. This means they will have an explicit agreement (not necessarily a signed MOU or contract, but at least well known to staff and agreed upon by clinic management) that patients from their clinic can go to a nearby health facility and obtain these tests within a reasonable delay. Reasonable is defined as the delay that would normally be incurred at health facilities that have these procedures on site. As well they must agree to perform contact investigations including Tuberculin skin testing and reading - if materials and training are provided.

We will select clinics that are representative of the diagnostic facilities and capacities available in clinics in the country. This means that clinics may be selected that have all diagnostic facilities on site, but others may be selected that do not have either chest-x-ray or GeneXpert, but have made administrative arrangements with nearby health facilities for the performance of these tests in their patients.

**Inclusion criteria**

Index TB patients:

1. New diagnosis of pulmonary microbiologically confirmed (smear, GX or culture) active TB within 30 days of treatment initiation.
2. Must have at least one identified household contact, and HHC investigation has not been started already.
3. Must agree to allow research team to access their medical history and approach their household contacts.

Household contacts:

1. Age 5-50 years.
2. On average in the past 3 months – slept in the same house, at least one night per week, or spent at least one hour per day for 5 days per week.
3. Pregnant woman can be included.
4. People with prior active TB or latent TB therapy will be included. These participants will be assessed for prevalent active TB, although they will not be treated for LTBI. Hence they will be included in the analyses of yield of active case finding, but excluded from analyses of numbers diagnosed and treated for LTBI.

**Exclusion criteria**

Index TB patients:

1. Known drug-resistant TB (INH resistant, multidrug resistance or rifampin resistance) may be excluded – after discussion in each country with TB program officials. If the TB programme’s policy is to screen contacts of MDR cases for active TB only and not provide any LTBI therapy, then index TB patients with MDR will be excluded as well as their HHCs. However, if the national TB program policy is to treat such individuals with standard LTBI therapy (since some HHCs of MDR patients will develop TB with drug-sensitive isolates later), then these index TB patients and their HHCs will be eligible. Hence, this will be a country-specific exclusion criterion.
2. Index TB patient with previous history of active TB (because their HHC may have undergone investigation before – which may change their need for study interventions, and also potentially change their perceptions and behaviours in the study).
3. Only has extra-pulmonary TB.
4. No identified household contacts.

Household contacts:

1. Members of the household, but do not meet the minimum time definitions for HH contacts.
2. Had TST/IGRA within 3 months.
3. People living with HIV. In most TB programs, HH contacts have unknown HIV status; HIV testing is recommended by WHO only if the index TB patient is known to have HIV co-infection. Contacts will be asked if they have been previously diagnosed to have HIV infection, and also asked if they are taking anti-retroviral therapy (if patients are receiving any medications, these will be checked carefully to verify what these are, and in particular if they are on anti-retroviral therapy). Both questions will be asked because some patients may be on therapy, but are not aware of the indication, or they may not wish to divulge their HIV status. If HHC are on anti-retroviral therapy and/or provide a history of previous HIV diagnosis, then they will be excluded, because the WHO recommended algorithm for investigation of household contacts who are HIV infected is different from that followed in the study arms. All index TB patients should undergo HIV testing based on national algorithm. If the index TB patient is found to be HIV positive, partner notification services will be recommended to the person living with HIV. The children of women who are HIV-infected should also undergo HIV testing. HIV testing will be offered to these household contacts (ie children and partners of an HIV infected index TB patient) who have not been HIV-tested within the last 6 months. If any household contact is found to be HIV-infected, they will be excluded pre-randomization. If there is a significant delay between identification of the household contacts and obtaining the HIV result, the HHCs can be randomized and then excluded post-randomization. These HHCs will be excluded from the modified intention to treat analysis, which will be the primary analysis. All HHCs identified to have HIV-infection will undergo investigations and treatment following national guidelines for HIV-infected household contacts.

4. If one household contact refuses to participate, then all HHCs will not be eligible. This is because we judge it infeasible to conduct one set of investigations for management of the consenting household contacts and a different set of procedures for management of HHCs that do not wish to participate. Hence, all household contacts must agree to participate and consent prior to enrolment. If a household contact is absent and will be away during the period of investigation, then we can proceed (e.g. household contact is traveling or will be away for extended time for work, etc.) but otherwise, we will attempt to enroll all HHCs, and if one or more do not agree, then we will not enroll any members of that household.

**Consent and enrolment**

The first step in enrolment will be to explain the study to all newly identified index TB patients with active pulmonary TB. Those that agree will provide the names of their HH contacts to study personnel, who will approach each HH contact (or their parent / guardian if a child aged 5-17 years) for individual signed informed consent. We will not seek informed consent from the index TB patients, since they do not actively participate in any study procedures. Members of the same HH do not have to all agree to participate, as the interventions are directed to each individual.

**Randomization**

If one or more HH contact agrees to participate and provides signed informed consent, then the first member of the HH will be randomized to one of three arms. All other consenting members of the HH will be assigned to the same arm, as long as they are enrolled within 14 days after the first HH member was randomized.

Randomization will be computer generated and stratified by randomization units within each country, in blocks of variable length. Randomization units will be defined on the basis of expected number of eligible index TB patients, and availability on-site of CXR facilities. Registration of participants, entry of information to verify eligibility, and randomization will be done via a web-based programme, that will be developed and housed by the Laboratoire de Télématique Biomédicale (LTB) du Réseau en santé respiratoire du Québec (RSR), with whom we have worked for similar data entry and randomization programs for more than a decade.

**SAMPLE SIZE CONSIDERATIONS**

LTBI treatment initiation:

We will base total sample size on the proportion of *identified* HH contacts who start LTBI treatment in each strategy. We are interested to detect a difference between the proportion of HH contacts starting LTBI therapy in those randomized to the standard algorithm and each of the alternative regimens. The proportion starting LTBI during phase 2 of ACT4, at the same study site, when solutions had been implemented and the barriers of TST and CXR had been resolved will be used as the likely proportion in the Standard arm. We consider that accepting to start treatment will likely be strongly influenced by other household members. We do not have an estimate of the cluster effect of HH on starting, so will use the “cluster effect” of study therapy completion which was observed in the 4V9 trial in the same sites. This gave an ICC of 0.33. Based on that and an average of three HH contacts aged 5-50 years (observed in ACT4), we can estimate the design effect as (1+household size)*ICC. We will not adjust for clustering at the level of the health facility, but instead will balance key characteristics within each country by stratifying randomization by characteristics of the health facility (CXR on-site), plus group facilities into randomization units of equal numbers of active TB patients.

**Table 1: Sample size required to detect superior initiation of LTBI therapy with either one of the experimental arms compared to standard arm**

| LTBI Initiation rate – among all HH Contacts identified | | Number required per group to detect significant difference*, accounting for clustering by household | | |
| --- | --- | --- | --- | --- |
| **Standard** | **Experimental (GX or noTST**)** | N per arm – 80% power | N per arm –60% power | Total N***  (3 arms) |
| 40% | 45% | 3046 | 1901 | 9138 |
|  | 50% | 766 | 478 | 2298 |
|  |  |  |  |  |
| 35% | 40% | 2921 | 1823 | 8763 |
|  | 42.5% | 1311 | 818 | 3933 |
|  | 45% | 742 | 464 | 2226 |
|  | 47.5% | 477 | 298 | 1431 |
|  |  |  |  |  |
| **30%** | 35% | 2734 | 1706 | 8202 |
|  | 40% | 703 | 439 | 2109 |
|  | **42.5%** | **455** | **284** | **1365** |
|  | 45% | 318 | 199 | 954 |
|  |  |  |  |  |
| 25% | 30% | 2484 | 1551 | 7452 |
|  | 35% | 649 | 405 | 1947 |
|  | 40% | 297 | 186 | 891 |

** alpha = 0.05. The intra-class correlation coefficient (ICC) or clustering effect of households on LTBI treatment initiation was estimated from the ICC for completion in the adult trial comparing 4R with 9H [16], among study subjects who had at least one other family member in the study – i.e. from participants in families of size >1. We expect the average number of household contacts to be 3 (based on our just completed ACT4 study).*

*** In the Standard and GX arms, all children <5 years, and older HH contacts who are TST positive will be eligible to initiate therapy. We estimate this will be about 50% of all HH contacts, resulting in the lower overall expected initiation rate among all HHC – as cannot exceed the expected proportion eligible for LTBI therapy. In the no TST arm we expect a higher proportion of HH contacts will start therapy, but we will estimate the number eligible based on prevalence of positive TST in the same age groups at the same centres in the other two arms; the number required in the no TST arm is therefore the same - based on this estimation.*

**** Total is based on 80% power.*

In the ACT4 trial, 60% of eligible HH contacts started an LTBI regimen once the TST and CXR problems were resolved. We assume that 50% of HH contacts will be eligible for LTBI therapy, and that 60% of eligible HH contacts in the Standard arm will start therapy – for an overall initiation rate of 30% with Standard arm in this trial. To detect an improvement such that 85% of eligible start therapy in the GX arm, for an overall initiation rate of 42.5% we would need to enrol **455** participants into each arm. Allowing for 5% withdrawal, or otherwise not analyzable participants, this would inflate the number per arm to 478, so we plan to enrol a total of **1434** participants. If the LTBI treatment initiation rate among eligible is 80%, providing an overall initiation rate that is only 10% better than Standard – then 1371 participants would still provide 60% power to detect a significant difference. Power will be greater if the initiation rate in the Standard arm is lower; for example, if only 50% of eligible HH contacts or 25% overall initiate therapy, as seen in Table 1.

Patient and health system costs:

Costs associated with the different study arms will be carefully measured. We expect that significant differences exist between sites in terms of costs – from perspective of health systems and patients. Hence, we have calculated study power for each country. For power calculations costs are based on estimates from the WHO CHOICE database and data gathered as part of prior studies (ACT4). As seen in Table 2, the number enrolled to each arm in each country should provide more than 90% power to detect a significant difference in costs.

**Table 2: Sample size required to detect significant difference in costs between Standard and GX arms – in each country**

| Estimated costs associated with Standard* CAD$ 2017 | | | Estimated costs associated with GX**  CAD$ 2017 | | | Power to detect effect sizes***  (Effect size = the detectable difference/SD) | | |
| --- | --- | --- | --- | --- | --- | --- | --- | --- |
| **Patient perspective^** | **Health System**  **perspective** | **Total** | **Patient perspective^** | **Health System**  **perspective** | **Total** | **0.3** | **0.33** | **0.5** |
| **Benin** | | | | | | | |  |
| 20 | 121 | 141 | 16 | 111 | 127 | 0.72 | 0.8 | 0.99 |
|  |  |  |  |  |  |  |  |  |
| **Brazil** | | | | | | | |  |
| 36 | 319 | 355 | 28 | 278 | 306 | 0.72 | 0.8 | 0.99 |
|  |  |  |  |  |  |  |  |  |

** For Standard Scenario:* *Assume that HH contact has two visits for TST (administration and reading). Half have three more visits for medical evaluation and CXR, and 20% of these have an added two more visits to collect sputum samples. 25% have all of the above, plus 1 visit for LTBI treatment initiation and 3 more visits for LTBI treatment follow-up.*

*** For GX scenario: Assume that HH contact has two visits for TST (administration and reading). Half have one more visit for medical evaluation and GX. One quarter also have one visit for LTBI treat initiation and 3 more visits for LTBI treatment follow-up.*

*^Costs from the Patient Perspective: Expenses associated with medical visits assumed to be $4.00 per visit in Benin, and $7.50 per visit in Brazil. This accounts for travel costs and additional expenses during travel or at medical visit.*

****To estimate power, we assume alpha = 0.05, and 455/3=152 analyzable subjects per group in each country, and we considered the effect size (detectable difference/SD). We do not know the standard deviation, but can estimate approximate costs, based on prior work in each country. As an example, based on costs collected previously in Ghana (neighbouring country to BENIN, that also participated in our prior RCT of 4RIF vs 9INH), we expect a difference in total costs of $28 between standard and GX arms. If the standard deviation is $84, then the effect size will be ($28/$84) = 0.33. After accounting for clustering by household, assuming an ICC of 0.33 and 4 subjects per household this effect size will result in estimated power of 80%. If the SD is actually smaller (SD=$56), then for the same expected difference in costs, we will have an effect size=0.5, providing 99% power to detect a significant difference.*

LTBI Treatment completion:

The power to detect a significant difference in treatment completion is determined primarily by the expected number who start LTBI therapy in each arm, and the expected completion rates in the Standard arm. The expected number who will start is taken from Table 1. In the 4R trial completion of 9H overall was 62% among adults, and 80% in children, compared to 75% and 90% completion rates for 4R respectively. Hence, we anticipate overall completion rates in these same sites with the Standard strategy to be similar – i.e. close to 70%. As seen below we will have more than 67% power to detect a significant difference in LTBI completion rates.

**Table 3: Power for various detectable differences in completion of LTBI therapy with GX or noTST arms** (numbers based on 455 analyzable subjects per arm, and initiation rates of 42.5% (GX or no TST arm) and 30% (standard arm), as shown in Table 1 above)

| LTBI completion rate (of those who start) | |  |
| --- | --- | --- |
| **Standard** | GX or noTST | Estimated Power |
| 50% | 60% | 0.26 |
|  | 65% | 0.52 |
|  | 70% | 0.78 |
|  |  |  |
| 60% | 70% | 0.29 |
|  | 75% | 0.57 |
|  | 80% | 0.83 |
|  |  |  |
| **70%** | 80% | 0.34 |
|  | **85%** | 0.67 |
|  | 90% | 0.92 |

** alpha = 0.05. The intra-class correlation coefficient (ICC) or clustering effect of households on LTBI treatment completion was estimated from the ICC for completion in the adult trial comparing 4R with 9H [16], among study subjects who had at least one other family member in the study – i.e. from participants in families of size >1. We expect the average number of household contacts to be 3 (based on our recently completed ACT4 study).*

Prevalent Active TB among HH contacts

In two systematic reviews, the prevalence of active TB among HH contacts, at the time of initial investigation, was 3-5% [19, 20]. We assume that any difference in detection of prevalent active TB will be the result of an increased number of HH contacts who complete the investigation algorithm, and not because of inherent differences in test characteristics (for example a difference in sensitivity between GX and CXR). Hence, extrapolating from Table 1, if the difference in proportion starting LTBI therapy is equivalent to the difference in the proportion completing the investigation – then we expect a difference that about 10% of HH contacts will complete the investigation algorithm with GX or CXR/no TST – which would mean a difference in detection of prevalent active TB of 10% of 5% or 0.5% or one more active case detected per 200 HH contacts enrolled. Given the anticipated enrolment and follow-up of only 455 per arm, it is unlikely that we will detect significantly more cases of active TB. However, we plan to maintain this as a secondary outcome – given the strong current global interest in methods to enhance active case detection. As well, power may be greater - if the prevalence of active TB is higher, or the differences between standard and experimental arms are greater than expected.

**DATA GATHERING:**

**Pre-randomization (baseline)**

For the index TB patient, we will collect information regarding demographics, results of AFB smear, GX, and/or cultures, and CXR.

For each HH contact identified, we will gather information regarding age, sex, co-morbid illnesses, relationship to the index TB patient, nights per week sleeping in the house or hours per week spent in the house. We will also gather information regarding prior history of tuberculosis, prior treatment for latent or active TB as well as any prior testing for tuberculosis, including TST or IGRAs.

**Post-enrolment and randomization - Investigation of HHC**

For consenting HH contacts, we will gather information on the investigations performed – including those mandated by the protocol (symptom screen, CXR or GX, TST or IGRA) and as well document all other investigations performed at the discretion of the treating team. One particular concern is the potential for errors made by the treating team in conducting investigations not mandated per protocol, due to their desire that HH contacts get certain tests even if not part of the strategy to which the HH was randomized. All investigations including dates performed and results will be recorded.

To measure one of the secondary outcomes, the sensitivity and specificity of CXR interpretation by the study site staff will be estimated from a second reading of a copy of the film (or digital file) by an independent reviewer. This reviewer will be a chest specialist with experience in TB, or a radiologist. The reviewer will judge if the CXR is normal or abnormal, and if abnormal - whether the abnormalities are consistent with active TB (yes/no) or suspicious enough for active TB to warrant further microbiologic investigations (yes/no). A fourth question will ask if there are additional respiratory diagnoses suggested by the CXR (yes/no). These finding will be used to estimate sensitivity and specificity of CXR reading by local staff. Missed minor abnormalities may result in commencement of treatment inappropriately in the CXR and standard arms. On the other hand, over-diagnosis (especially among children aged 5-10) also may have occurred. The CXR re-readings will not be done ‘in real time’ and the results will not be used clinically. However, results will be returned to the original CXR readers’ as a quality improvement measure.

**Follow-up during LTBI treatment**

If patients are recommended to take LTBI treatment by the treating team, this will be recorded and also whether the patient accepts and begins treatment. The definition of LTBI treatment started will be a prescription given or pills dispensed. Follow-up during LTBI treatment will not be mandated by protocol. This is a pragmatic trial, and the LTBI treatment is not part of the interventions to which participants are randomized. Hence, we cannot impose a burden on the participants, meaning that follow-up during LTBI treatment will follow local/national recommendations and norms, and that we will accept the treating team’s definitions of treatment completion or non-completion. As part of site staff training at the time of study initiation, we will review national and international recommendations for follow-up, as well as definitions of completion/non-completion, and seek a consensus among providers as to the optimal methods of follow-up for their site, that balance need for patient safety with feasibility and costs – for patients and the health system. All patient visits will be recorded and at these visits, we will request (or study staff will perform) pill counts.

To measure the secondary outcome of treatment completion, pills dispensed at each visit, as well as counts of pills remaining on return visits will be recorded and used to calculate treatment adherence and completion. Treatment completion will also be defined as having taken 80% of doses within 120% of allowed time; this definition will be used in secondary analyses.

**End of treatment – documentation**

End of treatment forms will be competed for all HHCs enrolled who start LTBI therapy. These will be completed when LTBI is stopped – either because treatment was completed (as defined above), or not completed – due to patient decision, provider decision due to adverse event, death, or other outcome.

**Health system costs and patient costs**

Differences in health system costs between the three strategies will focus on the costs related to the different tests being used, and related management of results of these tests. Each cost will be estimated using relevant cost components and an ingredients approach. The actual activities, investigations, supplies and services used for the investigation and treatment of all HHC, including follow up will be tabulated. Drug costs and all costs related to adverse event management will be included. The cost components required for each test are summarized in Table 4. Health personnel time is a key component of total test cost. The health personnel time for various LTBI management activities, including tuberculin skin test administration and reading, medical evaluation and initiation of treatment, will be taken from time and motion (TAM) studies completed during ACT4. The WHO CHOICE tool will be used to estimate health facility overhead costs and international suppliers such as Global Drug Facility (GDF) for the cost of materials where relevant (see below for specific details and references).

**Table 4: Cost components and potential sources of cost data for tests performed**

| Unit Cost and Components | Source / Method to Obtain / Data Collection Tool |
| --- | --- |
| TB Contact Investigation |  |
| HCW Time to Perform | TAMS and/or reported fees paid from ACT4 |
| HIV Test | From UNAIDS Agreement |
| Outpatient Visit | WHO-CHOICE tool – tailored to healthcare facility specifications |
| Patient Costs | Modified version of patient cost questionnaire geared toward LTBI |
| TB Symptom Screen |  |
| HCW Time to Perform | TAMS and median salaries from ACT4 |
| Outpatient Visit | WHO-CHOICE tool – tailored to healthcare facility specifications |
| Patient Costs | Modified version of patient cost questionnaire geared toward LTBI |
| Chest X-Ray |  |
| Cost of CXR and Personnel | Derived using an allocation key applied to healthcare facility overhead and personnel¶ |
| CXR-Related Patient Costs | The cost charged to the patient for the CXR (patient subsidizes CXR cost in Benin) |
| Non-CXR Related Patient Costs | Modified version of patient cost questionnaire geared toward LTBI |
| Interferon-Gamma Release Assay |  |
| Phlebotomist time to take blood | Collected through TAMS during study; median salary collected during study |
| Laboratory fee | Derived from healthcare facility fees |
| Outpatient Visit | WHO-CHOICE tool – tailored to healthcare facility specifications |
| Patient Costs | Modified version of patient cost questionnaire geared toward LTBI |
| Tuberculin Skin Test |  |
| Materials: syringe, tuberculin | Collected through ACT4 |
| HCW Time to Perform and Read | TAMS and median salaries from ACT4 |
| Two Outpatient Visits | WHO-CHOICE tool – tailored to healthcare facility specifications |
| Patient Costs | Modified version of patient cost questionnaire geared toward LTBI |
| Gene Xpert |  |
| Materials: Cartridge, Sputum Cup | From Global Drug Facility Diagnostics Catalogue |
| HCW Time to Take Sputum | Collected through TAMS during study; median salary from ACT4 |
| Laboratory Personnel Time to Run GX | TAMS and median salaries from ACT4 |
| Running Costs for Laboratory | Derived using an allocation key applied to healthcare facility overhead† |
| Outpatient Visit | WHO-CHOICE tool – tailored to healthcare facility specifications |
| Patient Costs | Modified version of patient cost questionnaire geared toward LTBI |
| Sputum AFB* |  |
| Materials: Sputum Cups | From Global Drug Facility Diagnostics Catalogue |
| HCW Time to Take Sputum | Collected through TAMS during study; median salary from ACT4 |
| Laboratory Personnel Time to Perform Smear | Collected through TAMS during study; median salary from ACT4 |
| Running Costs for Laboratory | Derived using an allocation key applied to healthcare facility overhead† |
| Outpatient Visit | WHO-CHOICE tool – tailored to healthcare facility specifications |
| Patient Costs | Modified version of patient cost questionnaire geared toward LTBI |
| Sputum Culture* |  |
| Materials: Sputum Cups | From Global Drug Facility Diagnostics Catalogue |
| HCW Time to Take Sputum | Collected through TAMS during study; median salary from ACT4 |
| Laboratory Personnel Time to Perform Culture | Collected through TAMS during study; median salary from ACT4 |
| Running Costs for Laboratory | Derived using an allocation key applied to healthcare facility overhead† |
| Outpatient Visit | WHO-CHOICE tool – tailored to healthcare facility specifications |
| Patient Costs | Modified version of patient cost questionnaire geared toward LTBI |
| LTBI Treatment (3HR, 3HP, 4R, 6H) |  |
| Materials: Drugs | From Global Drug Facility Medications Catalogue |
| Lab Monitoring: Blood Tests (LFT, HIV, etc.) | Derived from fees paid to local laboratory |
| HCW Time During Treatment Initiation and Follow-up (Physician, Nurse, Administrative) | Collected through TAMS during study; median salary from ACT4 |
| Adverse Event Management | Algorithm collected during study, with cost components collected throughout |
| Outpatient Visits | WHO-CHOICE tool – tailored to healthcare facility specifications |
| Patient Costs | Modified version of patient cost questionnaire geared toward LTBI |
| Active TB Treatment |  |
| Materials: Drugs | From Global Drug Facility Medications Catalogue |
| Lab Monitoring: Blood Tests (LFT, HIV, etc.) | Derived from fees paid to local laboratory |
| HCW Time During Treatment Initiation and Follow-up (Physician, Nurse, Administrative) | Collected through TAMS during study; median salary from ACT4 |
| Adverse Event Management | Algorithm collected during study, with cost components collected throughout |
| Outpatient Visits | WHO-CHOICE tool – tailored to healthcare facility specifications |
| Patient Costs | Modified version of patient cost questionnaire geared toward LTBI |

TAMS: Time and Motion Study; TB: tuberculosis

¶Chest x-ray is subsidized in Benin meaning the patient pays for a chest x-ray, but some of the cost is still covered by the healthcare system. To determine the healthcare system cost of CXR we will use a top-down approach with an allocation key derived based on proportion of manpower assigned to the radiology department who are required to perform CXR. The running costs of the radiology department would then be approximated by the proportion of healthcare facility overhead—excluding staff salaries (as they are already considered) and other unnecessary items for day-to-day function. Using the manpower allocation required to perform CXR, we can use median salaries of radiologists and radiology technicians to arrive at annual personnel related costs. We will sum the overhead and personnel costs and divide this value by the annual number of CXR performed at the facility to determine the cost per CXR performed.

†Allocation key derived based on proportion of manpower assigned to the laboratory compared to total manpower in the healthcare facility. The running costs of the laboratory would then be approximated by this proportion of healthcare facility overhead—excluding staff salaries (as they are already considered) and other unnecessary items for day-to-day function.

*For these unit costs, fees from the laboratory may be used as proxy measures of cost.

Key References for Table 4: GDF (<http://www.stoptb.org/gdf/>; WHO-CHOICE (<https://www.who.int/choice/cost-effectiveness/en/>) and Patient Cost Questionnaire; http://www.stoptb.org/wg/dots_expansion/tbandpoverty/assets/documents/Tool%20to%20estimate%20Patients'%20Costs.pdf

Patient costs must be carefully considered in LTBI management because this condition is asymptomatic, and treatment is for prevention of a disease that may never occur anyway. Willingness to pay is likely to be lower than for symptomatic conditions, and so patient out-of-pocket costs and time may be crucial barriers to successful completion of each step in the LTBI cascade of care. Patient direct and indirect costs will be measured for all household members; this includes time and travel costs for visits for investigation and treatment, and all out of pocket expenses, particularly for the time, travel and any other expenses related to the TST and CXR (even if the patients do not pay for the actual tests – these may require separate visits to the health facility just to complete them – and this requires time, and out-of-pocket expenditures).

We will adapt a standardized interviewer administered questionnaire developed used previously by us to measure patient and family costs associated with active TB [32-35]. We will include a small number of items on this questionnaire regarding patient experience and acceptability of study procedures. This questionnaire will be administered to patients in the third month post-randomization, meaning they will have undergone most investigations by that time. Hence, they should have a good perspective on their experience with tuberculin skin testing and reading, performance of CXR, and provision of sputum samples and performance of GeneXpert or microbiologic testing, while still being recent enough to avoid recall difficulties. (refer to appendix 5 for draft patient cost questionnaire)

**Data Analysis**

Modified Intention to treat: We will include all patients in this analysis, except for who were randomized but found to have exclusion criteria post-randomization – which may occur if index TB patients are found to have DR-TB, or HHCs are found to have HIV infection. Some HHCs may have additional of the three key tests (CXR, GX or TST) – that were not part of the algorithm to which they were randomized but were considered necessary by their provider. These HHCs will be included in the MITT analysis – according to the strategy to which they were originally assigned.

Per protocol analysis: This will include only HHCs who underwent the key investigations (CXR or GX or TST), according to the strategy to which they were randomized, but did not have other of the three investigations of interest in this study.

**Interim analyses/monitoring**

We will monitor for problems related to study investigations, as well as LTBI treatment. If differences in enrolment or drop-out rates between the arms are noted, we will investigate to ensure the site health care staff are not trying influence allocation of HH contacts to different arms, and that there are no cross-over problems – in which HH contacts randomized to one arm are investigated with an algorithm for a different arm. Early in the trial these may occur randomly due to errors caused by health system staff due to their understanding and application of the investigation algorithms in the three arms. Errors in the investigation algorithms that are detected will be reviewed as part of ongoing in-service training of the staff involved. If differences in enrolment to the 3 arms persist, we will investigate the potential reasons for this and provide further in-service training to reinforce study procedures.

**Final analyses**

Primary analysis

The primary outcome is the proportion starting LTBI therapy of those eligible (measured or estimated) for latent TB therapy. ‘Eligible’ will be defined as: aged 5-50 years, HIV uninfected and TST >5mm. For the no TST arm, the expected prevalence for HIV uninfected HH contacts aged 5 years and older will be estimated from age-specific prevalence of positive TST among HH contacts tested in the other two arms. This expected prevalence will be used to estimate the proportion ‘eligible’ for LTBI therapy in the no TST arm. Treatment initiation will be defined as being given a prescription for LTBI therapy, or dispensed the first month of pills needed for LTBI therapy. Since this is a dichotomous outcome, the primary analysis will be a logistic regression, using an identity link, and estimated via generalized estimating equations (GEE) to account for clustering by household. An exchangeable correlation structure and empirical standard errors will be used. We will compare the proportion starting LTBI treatment within 3 months of randomization of the contacts in each experimental arm against the standard arm.

Secondary analyses

1. Societal costs (health system and patient costs) of the full cascade of care - from initial identification to LTBI therapy completion will be considered. Using all relevant cost components (Table 4) and an ingredients approach, differences in health system costs will be assessed for each of the strategies. Patient time will be valuated based on an assumption of income equivalent to the average per capita income in the country. Health care personnel time will be valuated based on average salaries from information provided by facility management in each setting.

2. Prevalence of microbiologically confirmed and clinically diagnosed active TB – detected as part of the initial contact investigation, who initiate treatment within 3 months of the date of randomization of the contact, will be compared between all three arms.

3. Prevalence of positive TST (5 mm or 10 mm cut-points) by age group – 5-10, 11-17, 18-24, 25-34 years, and older in Standard and GX Strategies. This is simple descriptive analysis – and will be presented as overall prevalence in the specified age groups, plus stratified by country.

4. Incidence of serious adverse events related to LTBI therapy. Adverse events are relatively rare dichotomous outcomes. As such, Poisson regression will be used to compare the occurrence of the adverse events between each of the two experimental arms and the conventional arm. To account for clustering by household, we will use GEE, with an exchangeable correlation structure and empirical standard errors. We will compare in the same way - the occurrence of grade 1-2 adverse events reported by study investigators.

5. Completion of LTBI therapy –we will use the definition of completion/non-completion of the providers and the TB programs in each country, but in secondary analysis we will also define completion as taking at least 80% of doses in 120% of allowed time. (For 6H this means taking at least 144 doses within 216 days; for 4R this means 96 doses within 144 days, and for 3HR – 72 doses within 108 days.) Since this is a dichotomous outcome, the primary analysis will use logistic regression, with an identity link, and estimated via GEE to account for clustering by household. An exchangeable correlation structure and empirical standard errors will be used. We will compare the proportion completing treatment in each experimental arm against the standard arm.

6. Sensitivity and specificity of CXR reading by usual providers in each study site. For this analysis, the reference standard will be the readings by the external CXR review.

7. Active TB – defined as treatment initiated for active TB – detected only as a result of the CXR done in persons who could not produce a sputum sample.

**Ethical considerations**

The most important ethical considerations are the risks of treating undetected active TB with LTBI therapy and the risks of adverse events from LTBI therapy, particularly in participants who may not have latent TB infection.

To protect participants from inadvertent LTBI therapy of undetected active TB, we will follow guidelines from the WHO by using symptom screening, as well as CXR or GX screening in all HHC. GX is a sensitive test for detection of active TB, and several studies have demonstrated that GX can be used to replace CXR for detection of active TB in other populations. These studies suggest that GX will fail to detect only patients with minimal active TB, and in these persons, there is evidence that LTBI therapy is in fact adequate [28, 36]. In addition, we will review CXR independently, and provide quality assurance reports every quarter to CXR readers at each site. This feedback should help to enhance local proficiency in CXR reading, but if we detect substantial rates of missed diagnoses, then we will take corrective action in an ongoing fashion.

The issue of over-treatment of latent TB in study participants in the no-TST arm is addressed in detail in the rationale for interventions section above. Briefly, we are safeguarding the participants by undertaking the study in settings where almost 2/3 of household contacts have evidence of latent TB infection and so would likely receive LTBI therapy if they underwent TST. In addition, those who are most likely to be treated in the absence of LTBI are those aged 5-20 years as they have lower prevalence of positive TST [37, 38] but also least risk of adverse events from LTBI therapy. Additional safeguards are the use of the shorter regimens which will reduce burden, and risk of adverse events, and to exclude those aged over 50 years (although these persons could be tested and evaluated on an individualized basis only), as adverse events are more common in adults above this age limit.

This study will place additional burden on study participants, as with any research trial. However, we believe the benefits to participants outweigh the burden because all potential study participants are at high-risk of having active TB – either at the time of enrolment or within 2 years after. Participants in this this study should benefit from a reduced risk of morbidity and mortality from TB, besides the added benefit of reduced risk of further transmission within the household. All direct costs for study interventions (TST, GX or CXR) will be covered by the TB program, if usually covered, or by research funds. Therefore, study participants will not pay direct costs, such as for CXR. Other costs, and patient time for the health care facility visits for investigations or treatment would normally be incurred anyway. As well, these are important outcomes in their own right - high costs or significant loss of time would represent important barriers for future uptake of the results in these settings. Hence, patient out-of-pocket costs (e.g. for travel, or food), and time will be carefully measured. We will not compensate patients for these costs because this would potentially influence the study outcomes and are important outcomes for future generalizability of the study results.

All participants will provide signed informed consent, before randomization. For children aged 5-17 years, we will obtain parental signed consent, and for children aged 10-17 years, we will also ask the child to sign assent forms. This consent will include agreement that the digital file of the CXR can be stored in a de-nominalized database for later use to assess software for computer aided diagnosis of active TB. We will not ask index TB patients to provide consent, as they are not undergoing any study procedures, although they must agree verbally to allow research staff to approach their HH contacts. Individual members of each HH can decide independently to participate; it is not necessary for all HH members to participate together – as the study interventions are directed to each individual and not to the household itself.

This protocol as well as consent and parental/assent forms will be reviewed by an ethics review board of the RI of the MUHC. After approval by this board, it will be reviewed by research ethics committees at all participating centres.

**Trial management**

Scientific Advisory Committee (SAC)

The responsibilities of the SAC are to provide advice and recommendations regarding scientific aspects of the studies, particularly study design, interventions and outcomes as well as the statistical analysis plan. We have already formed a SAC for all projects within the CIHR Foundation grant.

Members of the SAC are: Dr. Hailey Getahun, responsible for anti-microbial resistance now, but until June 2018 was responsible for latent TB at the World Health Organization in Geneva, Switzerland; Dr. Bill Burman, director of Denver Public Health and long-standing member of TB Trials Consortium; Dr. Andy Vernon, director of Science, TB division, Centre for Disease Control, Atlanta, Georgia and director of TB Trials Consortium; Dr. Ben Marais, professor of University of Melbourne, Australia – considered the global authority on pediatric tuberculosis.

Data Safety and Monitoring Board (DSMB)

The DSMB will be responsible to review any unusual or unexpected events and make recommendations regarding continuing or stopping enrolment to study arms (or the overall study). They also will review on an ad-hoc and immediate basis any serious adverse events that are unexpected, as well as any deaths that are judged possibly or probably related to therapy of LTBI. Note that certain adverse events are *expected* as these occurred in our prior trials with 4R and 9H, and in published studies using the different LTBI regimens that may be used in this trial. These will not be brought to the attention of the DSMB when they occur, but will be included in all safety analyses, plus they are mentioned in the consent, and will be explained to the potential participants’ during the consent process. Ad-hoc reviews of *unexpected* serious adverse events will be whenever needed. DSMB recommendations following any ad hoc meetings will be sent to the research ethics committees at all participating sites.

Trial steering committee

The trial steering committee will review progress of the ongoing trial, including enrolment and randomization, pragmatic problems such as difficulties with enrolment or withdrawal of consent, as well as need for study amendments. The trial steering committee will also review recommendations of the SAC and the DSMB. The trial steering committee will be responsible for the final decision regarding stopping enrolment to any study arm, or the study. If such a decision is taken, the research ethics committees at all participating sites will be notified.

This committee will be comprised of the principal investigator (Dr. Menzies), the Foundation project manager (Dr. Oxlade), the trial biostatistician (Dr. Benedetti) as well as the principal investigators from each site (Benin and Brazil).

**REFERENCE**

1. Houben RM and Dodd PJ, *The Global Burden of Latent Tuberculosis Infection: A Re-estimation Using Mathematical Modeling.* PLOS Med, 2016. **13**(10): p. e1002152.

2. Dye, C., et al., *Trends in tuberculosis incidence and their determinants in 134 countries.* Bull. World Health Organ, 2009. **87**: p. 683-691.

3. Bucher, H.C., et al., *Isoniazid prophylaxis for tuberculosis in HIV infection: a meta-analysis of randomized controlled trials.* AIDS, 1999. **13**(4): p. 501-507.

4. Akolo, C., et al., *Treatment of latent tuberculosis infection in HIV infected person ( Review0.* The Cochrane Coolboration, 2010. **1**: p. 1-50.

5. World Health Organization, *Latent TB Infection : Updated and consolidated guidelines for programmatic management*. 2018: <https://www.who.int/tb/publications/2018/latent-tuberculosis-infection/en/>.

6. Rangaka, M.X., et al., *Predictive value of interferon-gamma release assays for incident active tuberculosis: a systematic review and meta-analysis.* Lancet Infect Dis, 2012. **12**(1): p. 45-55.

7. Auguste, P., et al., *Comparing interferon-gamma release assays with tuberculin skin test for identifying latent tuberculosis infection that progresses to active tuberculosis: systematic review and meta-analysis.* BMC Infect Dis, 2017. **17**(1): p. 200.

8. Abubakar, I., et al., *Prognostic value of interferon-gamma release assays and tuberculin skin test in predicting the development of active tuberculosis (UK PREDICT TB): a prospective cohort study.* Lancet Infect Dis, 2018. **18**(10): p. 1077-1087.

9. Sester, M., et al., *Risk assessment of tuberculosis in immunocompromised patients. A TBNET study.* Am J Respir Crit Care Med, 2014. **190**(10): p. 1168-76.

10. Garibaldi, R.A., et al., *Isoniazid-associated hepatitis. Report of an outbreak.* Am Rev Respir Dis, 1972. **106**(3): p. 357-65.

11. Kabbara, W.K., A.T. Sarkis, and P.G. Saroufim, *Acute and Fatal Isoniazid-Induced Hepatotoxicity: A Case Report and Review of the Literature.* Case Rep Infect Dis, 2016. **2016**: p. 3617408.

12. Alsdurf, H., et al., *The cascade of care in diagnosis and treatment of latent tuberculosis infection: a systematic review and meta-analysis.* Lancet Infect Dis, 2016. **16**(11): p. 1269-1278.

13. Ena J and Valls V, *Short-course therapy with rifampin plus isoniazid, compared with standard therapy with isoniazid, for latent tuberculosis infection: a meta-analysis.* Clin Infect Dis, 2005. **40**(5): p. 670-676.

14. Sterling TR, et al., *Three months of rifapentine and isoniazid for latent tuberculosis infection.* New Engl J Med, 2011. **365**(23).

15. Sterling TR, et al., *Flu-like and Other Systemic Drug Reactions Among Persons Receiving Weekly Rifapentine plus Isoniazid or Daily Isoniazid for Treatment of Latent Tuberculosis Infection in the PREVENt Tuberculosis Study.* Clin Infec Dis, 2015. **61**(4): p. 527-35.

16. Menzies, D., et al., *Four Months of Rifampin or Nine Months of Isoniazid for Latent Tuberculosis in Adults.* N Engl J Med, 2018. **379**(5): p. 440-453.

17. Diallo, T., et al., *Safety and Side Effects of Rifampin versus Isoniazid in Children.* N Engl J Med, 2018. **379**(5): p. 454-463.

18. Menzies D, et al., *Adverse events with 4 months rifampin or 9 months isoniazid as therapy for latent TB infection: results of a randomized trial.* Annals of Internal Medicine, 2008. **149**: p. 689-697.

19. Fox GJ, et al., *Contact investigation for tuberculosis: a systematic review and meta-analysis.* Eur Respir J, 2013. **41**: p. 140-156.

20. Morrison J, Pai M, and Hopewell P, *Tuberculosis and latent tuberculosis infection in close contacts of people with pulmonary tuberculosis in low-income and middle-income countries: a systematic review and meta-analysis.* Lancet Infect Dis, 2008. **8**: p. 359-368.

21. Tanimura, T., et al., *Financial burden for tuberculosis patients in low- and middle-income countries: a systematic review.* Eur Respir J, 2014. **43**(6): p. 1763-75.

22. Wingfield, T., et al., *Defining catastrophic costs and comparing their importance for adverse tuberculosis outcome with multi-drug resistance: a prospective cohort study, Peru.* PLoS Med, 2014. **11**(7): p. e1001675.

23. Balcells, M., et al., *Isoniazid preventive therapy and risk for resistant tuberculosis.* Emerg Infect Dis, 2006. **12**(5): p. 744-751.

24. Samandari, T., et al., *A randomized placebo-controlled trial of 6 versus 36 months Isoniazid tuberculosis preventive therapy for HIV-infected adults in Botswana.* Lancet, 2011. **377**: p. 1588-1598.

25. Getahun, H., et al., *Development of a Standardized Screening Rule for tuberculosis in People Living with HIV in Resource Constrained Settings: Individual participant Data Meta-analysis of Observational Studies.* PLOS Medicine, 2011. **8**(1): p. e1000391.

26. van't Hoog, A.H., et al., *Screening strategies for tuberculosis prevalence surveys: the value of chest radiography and symptoms.* PLoS One, 2012. **7**(7): p. e38691.

27. Pedrazzoli, D., et al., *Can tuberculosis patients in resource-constrained settings afford chest radiography?* Eur Respir J, 2017. **49**(3).

28. Mount, F.W. and S.H. Ferebee, *Preventive effects of isoniazid in the treatment of primary tuberculosis in children.* N Engl J Med, 1961. **265**: p. 713-21.

29. Corbett EL, et al., *Comparison of two active case-finding strategies for community-based diagnosis of symptomatic smear-positive tuberculosis and control of infectious tuberculosis in Harare, Zimbanwe (DETECTB): acluster-randomised trial.* Lancet, 2010. **376**: p. 1244-1253.

30. Kopanoff DE, Snider DE, and Caras GJ, *Isoniazid-related hepatitis.* Am Rev Respir Dis, 1978. **117**: p. 991-1001.

31. Salpeter, S.R., et al., *Monitored isoniazid prophylaxis for low-risk tuberculin reactors older than 35 years of age: A risk-benefit and cost-effectiveness analysis.* Ann Intern Med, 1997. **127**(12): p. 1051-1061.

32. R, S., et al., *Patients costs and cost -effectiveness of tuberculosis tretment in DOTS and non-DOTS facilities in Rio de Janeiro, Brazil.* Plos ONE, 2010. **5**(11): p. e14014.

33. Rouzier, V.A., et al., *Patient and family costs associated with tuberculosis, including multidrug-resistant tuberculosis, in Ecuador.* Int J Tuberc Lung Dis, 2010. **14**(10): p. 1316-1322.

34. KR, J., et al., *Costs Incurred by Patients with Pulmonary Tuberculosis in Rural India.* Int J Tuberc Lung Dis, 2009. **13**(10): p. 1281-1287.

35. Aspler A, et al., *Cost of tuberculosis (TB) diagnosis and treatment from the patient perspective in Lusaka, Zambia.* Int J Tuberc Lung Dis, 2008. **12**(8): p. 928-935.

36. Ferebee SH, *Controlled chemoprophylaxis trials in tuberculosis.* Adv Tuberc Res, 1969. **17**: p. 28-106.

37. Menzies, R., *The determinants of the prevalence of tuberculous infection among Montreal schoolchildren, Thesis for M.Sc. in Epidemiology and Biostatistics*. 1989, Montreal: McGill University.

38. Menzies, R., B. Vissandjee, and D. Amyot, *Factors associated with tuberculin reactivity among the foreign-born in Montreal.* Am Rev Respir Dis, 1992. **146**: p. 752-756.
