# Supplementary material for: Rapid molecular testing or chest X-ray or tuberculin skin testing for household contact assessment of tuberculosis infection: A cluster-randomized trial
Source: PLoS Med. 2025 Jul 28;22(7):e1004666. doi: 10.1371/journal.pmed.1004666 (PMC12316388; doi:10.1371/journal.pmed.1004666)
Supplement: S1 Statistical Analysis Plan — (DOCX) [file pmed.1004666.s005.docx]

**13 Jan 2023 STATISTICAL ANALYSIS PLAN**

**GXT – GeneXpert or chest-X-ray or Tuberculin skin testing for household contact assessment: a cluster randomized trial**

Dick Menzies, Mayara Lisboa Bastos, Chantal Valiquette, Andrea Benedetti

**Primary objective:**

To compare the proportion of participants that started tuberculosis preventive therapy (TPT) within 3 months of randomization, between three different strategies for management of HIV uninfected household contacts (HHC) aged 5-50 years old of persons with newly diagnosed microbiologically confirmed active pulmonary TB.

**Secondary objectives**

To compare the following outcomes between three different strategies for the management of HIV uninfected HHC aged 5-50 years of persons with newly diagnosed microbiologically confirmed active pulmonary TB:

i) prevalence of active TB (confirmed and clinical separately) detected within 3 months after randomization;

ii) time from randomization to TPT initiation,

iii) TPT related outcomes of completion and adverse events,

iv) total societal costs (health system and patient costs)

v) incidence of active TB – notified to the National TB programme up to 6 months after the last participant was randomized.

**Study Population**

HIV uninfected household contacts (HHC) aged 5 to 50, of patients with newly diagnosed microbiologically confirmed pulmonary TB.

**Sample Size**

Table 1- Sample Size calculation in original protocol

| **LTBI Initiation rate – among all HH Contacts identified** | | **Number required per group to detect significant difference (alpha 0.05), accounting for clustering by household** | | |
| --- | --- | --- | --- | --- |
| **Standard** | **Experimental (GX or no TST**) | **N per arm – 80% power** | **Total N (3 arms)** | **Accounting for 5% withdrawn** |
| **30%** | 35% | 2734 | 8202 | 957 |
|  | 40% | 703 | 2109 | 738 |
|  | **42.5%** | **455** | **1365** | **478** |
|  | 45% | 318 | 954 | 334 |

We estimated that 50% of HH contacts would be eligible for TPT, and that 60% of eligible HH contacts in the standard arm (strategy 1) would start TPT – for an overall TPT initiation rate of 30% in HHC randomized to the standard arm. To detect an improvement such that 85% of eligible start therapy in the GX arm, for an overall initiation rate of 42.5% we would need to enrol 455 participants into each arm. Allowing for 5% withdrawal, or otherwise not analyzable participants, would inflate the number per arm to 478, so we planned to enrol a total of 1434 participants, of whom 1000 would be enrolled in Benin and 434 in Brazil.

**Changes to enrollment targets**

During monitoring visits in Benin we observed that strategy completion rates were much higher than under routine conditions. One possible explanation was that travel was reimbursed for ALL trial-related visits. Under routine conditions, the Benin National TB program reimburses travel costs just for an initial evaluation visit of HHC, and no further reimbursement is provided. Since the trial was intended to simulate program conditions, as much as possible, after January 1, 2022, participants in Benin only received the reimbursement for the first trial visit, as this is what is done under routine program conditions. In Brazil, no reimbursement for travel expenses is provided by the TB program under routine conditions, so no incentives or enablers (e.g., reimbursement of travel expenses) were to be given.

To be able to assess whether this reimbursement affected study outcomes, in January 2022 we increased the recruitment target from 1,000 to 1,250 in Benin so that approximately half of participants from Benin received incentives, and half did not. Enrolment of 1,273 participants was completed on 30 August 30, 2022; 624 HHC received incentives, and 649 did not receive any incentives.

In Brazil, our original sample size was 434 HHC. Due to the effect of the COVID-19 pandemic in Brazil, leading to health system disruption, recruitment in Brazil was significantly delayed. The original sample size was inflated by extra 5% to account for post-randomization exclusions, or otherwise non-analyzable participants. However, by August 2022, only 7 participants (0.4%) were excluded after randomization or were non-analyzable for other reasons. Since the magnitude of loss was so much less, we downsized our target recruitment to 370 participants in Brazil. Enrolment was stopped at the end of October 2022, due to lack of funding (staff had to be paid during COVID pandemic even though no study related work was possible).

**Definitions of the Strategies**

Strategy 1: Standard. This strategy is based on the current World Health Organization recommended algorithm for HHC contacts that are HIV negatives and age > 5 years. HHC undergo to symptoms screen and tuberculin skin test (TST). If either TST or symptom screen are positive (cough, sputum, fever, anorexia/weight loss), participants will undergo a chest x-ray (CXR). If CXR abnormal possible active TB, additional microbiologic test can be requested.

Strategy 2: Rapid molecular test (RMT – in this study all used GeneXpert) replaces CXR. HHC undergo to symptoms screen and TST. If either TST or symptom screen are positive (cough, sputum, fever, anorexia/weight loss), participants will undergo a RMT.

Strategy 3: No TST. HHC undergo CXR regardless of presence of symptoms. If the CXR is abnormal, or CXR normal and symptoms then microbiologic investigations will be done.

**Data Cleaning & checks**

We will perform descriptive analyses of all variables to detect possible data entry errors, and to detect outliers. For instance, we will verify all continuous variables' minimum, maximum, and mean values. Histograms will be constructed to examine the distribution of important variables such as age. The distributions of binary and categorical variables will be examined.

**Missing Data**

A complete case approach will be followed given that we anticipate complete data for most subjects.

**FINAL ANALYSES**

**1 - Variables**

Independent variables.

Our independent variable is the strategy to which the patient is randomized. We will compare the outcomes in each of the two experimental arms against the standard arm.

Covariates

Country, Age as a continuous variable (In sub-analyses we may examine outcomes by arm within categories of age such as: 5-18, 18-35, 36-50), Sex, Body size: Height (median, 25th, 75th percentiles), Weight (median, 25th, 75th percentiles), BMI (WHO categories). Other risk factors for developing active tuberculosis (TB) that might impact participants’ decision to complete the strategies and start tuberculosis preventive therapy (TPT): Diabetes, Renal Failure, solid organ transplant, use of TNF alfa inhibitors, other immunosuppression conditions or immunosuppressive therapy. Lifestyle behaviour: smoking (categorized as current smoker, former smoker, never smoked), Alcohol consumption (categorized as less than once a month, once a week, 2-3 times per week, four or more times per week). Random intercept in GLMM.

**2 - Population analyzed**

Modified Intention to Treat (MITT): We will include all participants in this analysis, except for those who were randomized but found to have exclusion criteria post-randomization – which may occur if index TB patients are found to have MDR-TB or HHCs are found to have HIV infection, or pregnant (only in Brazil).

**3 - Descriptive analyses**

We will compare participants randomized to the three strategies on the basis of: age, sex, BMI, smoking status, alcohol consumption, and comorbidities (renal Failure, Transplant, use of TNF alfa inhibitors, other immunosuppression conditions, and other immunosuppressive therapy – may be combined if small numbers as expected, or each condition if adequate numbers).

We will describe the crude proportions with primary, and secondary outcomes described below (overall, per country, and per strategy):

**4 - Analysis for Primary Objective**

Primary outcome:

The primary outcome is the proportion starting TPT of those eligible for latent TB therapy within three months of randomization. For strategy three, we will analyze these two ways: 1) the proportion starting TPT of all HHC randomized to this strategy and 2) the proportion of TPT-eligible who start TPT (this is also termed ‘probable TB infection’, and will be estimated by extrapolating from the country- and age-stratified TBI prevalence in Strategies 1&2.).

TPT started will be defined as a prescription given or pills dispensed. TPT-Eligible will be defined according to the strategy: Strategy 1 and 2: HHC with TST >5mm. For Strategy 3, the number who are TPT-eligible will be estimated in two ways: i) All HHC randomized to this strategy; and, ii) based on estimated TBI prevalence using the age-specific prevalence (calculated separately for Benin and Brazil) from the other two arms.

Since this is a dichotomous outcome, we will fit an unadjusted logistic regression model, using an identity link, and estimate via generalized estimating equations (GEE) to allow us to take clustering by Household, and site into account. An exchangeable correlation structure and empirical standard errors will be used. We will compare the proportion starting TPT within 3 months of randomization of the contacts in each experimental arm against the standard arm. The outcome will be expressed as risk-difference. No further adjustment will be performed, as this analysis follows the randomization .

Sensitivity analyses

We aim to assess the effect of visits reimbursement on TPT initiation, using a stratified analyses comparing the TPT initiation among those that received, or did not receive reimbursement. For Benin we will define that all participants randomized after 1 January 2022 did not receive any reimbursement. A similar model described above will be fit, but adjusted for participants’ demographic and clinical characteristics, as we are breaking the randomization.

**5 – Analysis for Secondary Objectives**

5.1 - Strategies ‘completed’ and ‘completed correctly’ (per protocol) :

We will assess the acceptability of the three strategies and TPT by providers and patients by comparing proportion of participants in each strategy who Complete, and complete correctly the strategy to which they are randomized.

We define a strategy as completed if participants underwent all investigations that were specified in the study protocol for that strategy within three months after randomization. We define a strategy as “completed correctly” if participants underwent all specified investigations and none other (i.e., no additional tests). For example: Strategy 2 Completed correctly: would mean a TST was done; if positive, a GeneXpert was done and if asymptomatic and GX negative, no further tests. Strategy 2 Completed but not correctly: TST done and if positive, a GeneXpert done. If asymptomatic and GX negative, a chest x-ray was done (CXR not part of the Strategy.

Completion and correct completion of strategies are not random events and may be associated with characteristics associated with the risk of TB disease and/or providers’ preferences and beliefs. For example, providers might request additional tests if they consider participants to have a higher risk of active TB. We will, therefore, fit an adjusted binomial regression model using an identity link and estimate via GEE accounting for HHC clustering. An exchangeable correlation structure and empirical standard errors will be used. The dependent variables are dichotomous (completed vs. did not complete strategy, or did/didn’t complete correctly), the independent variable is the strategy, and participants’ demographic and clinical characteristics and country will be included in the model as co-variates.

In secondary analyses, we will conduct stratified analyses comparing the completion of strategies among participants who did, or did not, receive incentives. To categorize participants, we will define that all participants in Benin randomized before 1 January 2022 received incentives, and all participants from Manaus randomized before 10 August 2022 received incentives. Participants randomized after those dates in the two settings (and all other participants from Brazil) will be defined as NOT receiving incentives. Same model described above will be fit.

5.2 – TPT completion

TPT completion will be defined as took at least 80% of prescribed doses, regardless of time. Risk differences will be estimated by comparing strategies 2 and 3 against the standard arm (strategy 1). A similar model as described above (for strategy completeness) will be fit, with the TPT regimen as a co-variate. TPT regimen will be categorized in different ways depending on the number of participants receiving each regimen. For instance: short rifamycin based regimens vs long regimens, or rifamycin (4R, 3HP and 3HR) compared to each other. We will conduct the same stratified analyses comparing the completion of TPT among those who received or not incentives (see section above)

5.3 – TPT-related AE that led to drug discontinuation:

The occurrence of an adverse event is not a random event, as the choice of TPT regimen was the provider’s decision. Therefore, providers might choose regimens with better safety profiles for patients they judged at greater risk for AE. Consequently, we will fit an adjusted binomial regression model using an identity link and estimate via GEE accounting for HHC clustering. An exchangeable correlation structure and empirical standard errors will be used. The dependent variables will be considered dichotomous (occurrence or not of AE), the primary independent variable is the strategy, and covariates will include participants’ demographic and clinical characteristics, country and TPT regimen used.

5.4 Confirmed and clinical active TB

*5.4.1 – Prevalent TB Disease*.

The prevalence of microbiologically confirmed, or clinically diagnosed active TB, defined as TB disease detected as part of the initial contact investigation, within three months of the date of randomization will be compared between the three strategies.

This will be a descriptive analysis, as our sample size was not calculated to detect a difference in prevalent active TB between the three strategies. Because clinical TB based on CXR interpretation may be inaccurate and even potentially biased, we will stratify into two groups: 1) microbiologically confirmed and 2) clinically confirmed.

*5.4.2 – Incident TB Disease:*

An amendment to the original protocol, is the addition of ascertainment of incidence of active TB among participants that is reported (notified) to the National TB program in the two countries. This was added to ensure that persons who dropped out, did not take TPT, or, in the GX arm – had active TB, but were not detected because they could not produce a sputum sample. For pragmatic reasons, incidence for all participants in each country will be ascertained at one time, which will be on the date (or soon after) which is six months after the last participant was enrolled (ie in early March 2023 in Benin where enrolment ended August 31, 2022, and early May 2023 in Brazil where enrolment ended October 31, 2022). We will use person time analysis to account for the variable length of follow-up from randomization to ascertainment of notification. Follow-up will be estimated as the time from randomization to the date when notifications are reviewed by the National TB program personnel. Incidence, per 100 person years of follow-up, will be compared between the three arms. This is a descriptive analysis only, as number of persons notified with TB are expected to be low.

**5.5 Societal costs**

We have planned a cost-effectiveness analysis – comparing costs per person initiating TPT by arm. In the event there is no difference in the outcome of persons initiating TPT between arms, we also plan a cost minimization analysis. The primary analysis is a comparison of societal costs (health system plus patient) by strategy, but we will also compare the two major components of health system and patient costs by strategy.

5.5.1 Health System Costs

*Tabulation of health care activities*:

Using the data collected from our CRFs, we will first tabulate the healthcare use by participants. We will include all activities related to investigations, supplies, medications, testing, and services used to investigate and treat all HHC, including follow-up and management of possible adverse events or active tuberculosis. Table 2 describes possible activities gathered from CRFs.

*Costing each health care activity*:

Then, after the tabulation of activities per participant, we will value those activities. We will obtain local unit costs from each site, Brazil and Benin. For tests, we will use information from previous recent studies published in 2020^1^ and 2021^2^. The costs of routine follow-up visits and the per diem hospitalization costs for all sites were taken from the World Health Organization's CHOosing Interventions that are Cost Effective tool (WHO CHOICE tool)^3^. Prices of TPT and active TB drugs will be taken from the price list of the Global Drug Facility. In sensitivity analyses we will use drug prices paid by the Ministry of Health in Brazil, for Brazil health system costs

Health personnel time is a key component of total health system cost. The health personnel time for various TBI management activities, including tuberculin skin test administration and reading, medical evaluation and initiation of treatment, will be taken from time and motion (TAM) studies completed in a previous study by our research group^4^. Health time personal will be valuated based on average salaries from information provided by facility management in each setting in 2022

Table 2- Health system costs - Activities components and possible personal time allocated to each of those activities.

| **Health System Activities** | **Personnel time** | **Materials/Other** |
| --- | --- | --- |
|  |  |  |
| **Investigation period** | |  |
| *Outpatient visits per participant* | Time for HCW to conduct HHC investigation | Transport to patient home (if home visit) |
| *Tests done per participant* |  |  |
| Tuberculin skin test | HCW Time to Perform and Read (taken from a TAM study in ACT4 – average for LMIC will be used) | syringe, gloves, alcohol swab, tuberculin |
| AFB | HCW Time to Take Sputum/ Laboratory Personnel Time to run AFB | AFB materials (slide, staining supplies, microscope), sputum cup --- consider maintenance and calibration; overhead of lab |
| Sputum culture | Laboratory Personnel Time to Perform Culture | culture materials (bactec, supplies – reagents and consumables) --- consider maintenance and calibration; overhead of lab |
| GeneXpert | HCW Time to Take Sputum/ Laboratory Personnel Time to Perform xpert | cartridge, maintenance, calibration, machine, lab overhead |
| Chest x ray | HCW time to request CXR/Technician time to perform CXR/time to interpret cxr | xray films (analog), cost of machine, radiology overhead, |
| Liver function tests | Phlebotomist time to take blood/ HCW time to run the tests | lab fees for LFT |
| Red blood cells/White blood cells | Phlebotomist time to take blood/ HCW time to run the tests | lab fees for CBC |
| Other tests requests by HCW | HCW time to run the tests | variable |
| **Follow up visit** | |  |
| Outpatient visits per participant | HCW Time During Follow-up (Physician, Nurse, Administrative) | WHO CHOICE |
| *Tests done per participant* |  |  |
| Red blood cells/White blood cells | Phlebotomist time to take blood/ HCW time to run the tests | as above |
| Liver function tests | Phlebotomist time to take blood/ HCW time to run the tests | as above |
| Microbiologic tests (for further active TB investigation) | HCW Time to Take Sputum/ Laboratory Personnel Time to run tests | as above |
| Image tests (for further active TB investigation) | HCW time to request CXR/Technician time to perform CXR | as above |
| *Drugs* |  |  |
| TPT regimen doses taken | HCW Time to dispense drugs | cost of drugs (in specific dose combinations by weight, if children); cost of adjunct medication, if needed |
| Other medication used to manage possible TPT intolerance |  | medication costs |
| **Adverse event** | |  |
| *Outpatient visits per participant* | HCW Time During Follow-up (Physician, Nurse, Administrative) | WHO-CHOICE |
| *Tests done per participant* | HCW Time to dispense drugs | based on tests |
| *Hospitalization days* | *-* | per diem by day |
| *Drugs to manage the AE* | *-* | medication costs |
| **Active TB management** | |  |
| *Outpatient visits per participant* | HCW Time During Follow-up (Physician, Nurse, Administrative) | WHO-CHOICE |
| *Tests done* |  |  |
| Red blood cells/White blood cells | Phlebotomist time to take blood/ HCW time to run the tests | as above |
| Liver function tests | Phlebotomist time to take blood/ HCW time to run the tests | as above |
| Microbiologic tests | HCW Time to Take Sputum/ Laboratory Personnel Time to run tests | as above |
| Image tests | HCW time to request CXR/Technician time to perform CXR | as above |
| *Drugs* |  |  |
| Doses Active TB regimen (RZHE) | HCW Time to dispense drugs | 6HR/2ZE at appropriate doses by weight |
| Other medications related to active TB treatment | - | Medication costs (eg, pyridoxine) |
| Hospitalization days | *-* | *per diem by day from WHO-CHOICE* |

Costing data will be collected in local currency and then inflated using local inflation indices to 2022, if necessary. These calculations will be then exchanged to US dollars using either direct exchange rates (for tradable items, including tests and latent tuberculosis drug) or purchasing power parity exchange (for salary and non-tradable items).

*Calculating each participant’s health system costs:*

For each study participant, the number of times each health care service was used will be multiplied by the unit cost of the activity. For instance, if a patient had two follow-up visits, the health system costs for that type of activity will be calculated:

*2 x (unit cost of outpatient visit + valuated time spent by HCW to perform the visit)*

Then, all activity costs will be summed to provide a total health system costs per participant.

*Comparing the three strategies*:

The average (mean/median) per participant health system costs will be compared between the three strategies, using an approach we have recently used (*Bastos, Annals Int Med*). Specifically, we will calculate a ratio of mean costs per participant assigned to strategy 2, or to Strategy 3, divided by the mean cost per participant assigned to strategy 1, to provide a summary estimate of the health system costs for the experimental strategies relative to the standard strategy. By using a ratio of costs, we can combine results from the two countries, despite large differences in absolute costs. Each participant’s total health system costs will also be used to calculate the total and mean cost for participants who met different study end points (TPT initiation, completion, Active TB diagnosed or SAE), although this will have to calculated by country.

5.5.2 - Patients Costs

*Tabulating patents’ activities and time:*

A questionnaire was administered to HHC in each country (120 in Benin and 76 in Brazil), with roughly equal numbers per strategy. This was administered between one to three months post randomization in order to capture information on costs related to investigation and treatment (if any). Only one HHC per family was interviewed. We collected information on out-of-pocket direct costs for visits, travel, and tests, as well as time spent on travel, visits and performing tests (indirect costs). For children, we collected information on the care-givers’ time as well; this time will be used to estimate indirect time for the care of the children.

*Valuating activities and time:*

Indirect costs will be valued based on information on the type of work performed by the HHC, and average salaries for that work in each county using national data (from Benin and Brazil).

*Calculating total patient costs – per participant in the PCQ:*

We will sum each patient indirect + direct costs as an overall total patient cost. For each study participant we collected information on their direct and indirect costs for each activity (e.g., a visit to the health centre). Because the questionnaire was administered once during the first three months post-randomization, the total number of times each activity was performed was not always known at the time of the interview. However, the interviews were planned to be conducted at about three months post randomization, so the diagnostic investigations should have been completed and TPT initiated in those eligible. TPT visits are close to identical after the initial 'start' visit, so we will extrapolate costs of follow-up based on the time and activities during the first follow-up visit. Total patient costs for each activity will be multiplied by the number of times each visit occurred in that participant (taken from CRFs completed by study staff). For instance, if a patient had two follow-up visits: *2 x (Direct costs related to a FU visit + Indirect costs related to a FU visit*).

Then, all activity costs will be summed to provide a total cost per PCQ participant.

We will first calculate total patient costs among participants who completed the PCQ. Some participants will only have diagnostic procedures, and hence contribute to estimating these costs, while others will also have TPT, and so will contribute to estimating diagnostic and treatment costs. Among people in each arm contributing data to the diagnostic period we will calculate average diagnostic costs, and among people contributing data to treatment, we will calculate average treatment costs. Then we will develop a model to predict costs among participants in whom these patient costs are not known, based on arm, whether they had diagnostic procedures alone, or diagnostic and treatment, plus patient characteristics significantly associated with patient costs (We will evaluate in univariable analysis, and include in this predictive model all variables with p<0.2. Alternatively we could include characteristics simply on the basis of prior evidence these are important (sex, employment, household size, age, etc).

*Comparing patient costs:*

For total, as well as direct and indirect costs, we will calculate a ratio of mean costs per participant randomly assigned to strategy 2 or to Strategy 3 divided by the mean cost per participant assigned to strategy 1. This will provide a summary estimate of the patient’s relative costs (total, direct and indirect) for the three strategies and allow us to combine data from the two countries (as ratios). We will also use the calculated total costs per participant to calculate the total and mean cost for participants who met different end points (TPT initiation, TPT completion, Active TB diagnosed or AE).

There are scarce data on published patient’s costs related to TBI investigation and treatment. Therefore, we plan to report detailed analyses on patient’s costs in a separate publication.

5.5.3 - Societal costs

Total societal costs per participant will be the sum of health system and total patient costs.

Health system costs will be known for all participants in the study based on their documented health system activities in the study. Patient costs will be predicted for the study participants who did not complete the PCQ, based on the results from the sample of HHC who did complete the PCQ, as described above. This will be used to calculate individual societal costs for all 1600 participants, providing more robust estimates for analyses of primary, and secondary endpoints.

References

1. Bastos ML, Campbell JR, Oxlade O, et al. Health System Costs of Treating Latent Tuberculosis Infection With Four Months of Rifampin Versus Nine Months of Isoniazid in Different Settings. *Ann Intern Med* 2020; **173**(3): 169-78.

2. Oxlade O, Benedetti A, Adjobimey M, et al. Effectiveness and cost-effectiveness of a health systems intervention for latent tuberculosis infection management (ACT4): a cluster-randomised trial. *The Lancet Public health* 2021; **6**(5): e272-e82.

3. Bertram MY, Lauer JA, Stenberg K, Edejer TTT. Methods for the Economic Evaluation of Health Care Interventions for Priority Setting in the Health System: An Update From WHO CHOICE. *International journal of health policy and management* 2021; **10**(11): 673-7.

4. Alsdurf H, Oxlade O, Adjobimey M, et al. Resource implications of the latent tuberculosis cascade of care: a time and motion study in five countries. *BMC Health Serv Res* 2020; **20**(1): 341.
