# Supplementary material for: Rapid molecular testing or chest X-ray or tuberculin skin testing for household contact assessment of tuberculosis infection: A cluster-randomized trial
Source: PLoS Med. 2025 Jul 28;22(7):e1004666. doi: 10.1371/journal.pmed.1004666 (PMC12316388; doi:10.1371/journal.pmed.1004666)
Supplement: Attachment — Submitted filename: PMEDICINE-D-24-03440R1_Velen.docx [file pmed.1004666.s007.docx]

Abstract:

1. No changes suggested

Introduction:

1. Update reference to the Global TB report as the 2024 version is now available.
2. It would be helpful to expand the reasoning for WHY/HOW was the TPT targets achieved among PLHIV, but not for other groups?
3. In paragraph 2, there is mention of WHO not requiring the use of TBI testing or CXR, but TPT started based on risk group classification – this sentence is misleading, as the guidance still requires ruling out TBD or at least not having clinical suspicion for TB.
4. Use of PLHIV and PHIV – be consistent with abbreviations.

Methods:

1. Within the definition of index patients, the abbreviation for GeneXpert is used i.e. GX; please spell this out first before using the abbreviation. Also, clarify if this was GeneXpert MTB/Rif or Ultra.
2. Among index patients, how was drug resistance ruled out among those diagnosed on smear or culture – was additional reflex testing done to rule out such patients as having additional drug resistance; please clarify.
3. Among those HHC with a known HIV status (they were not tested by the study), how was the status confirmed; this is limitation since some (with an unknown status) would have been tested while the ‘other group’ would have been largely self-reported.
4. As described within the ‘Standard’ intervention, was CXR read once (and a decision made) or were multiple reads conducted; it is assumed that this was done by human-readers and not aided by computer-aided detection software, however this should be explicitly stated. In addition, please clarify what microbiological tests were done.
5. Was the same TPT used in all interventions? Currently this is described within the ‘Experiment 2’ paragraph, which suggests it only applied to this intervention; might help to have this separated if applied to all interventions.
6. In the data analysis, treatment initiation was defined as receiving a prescription, or dispensed medications for TPT – these are two quite different prospects in relation to ‘initiating treatment’ i.e., pills in hand are close to initiation treatment versus having to still collect medication; is there a reason why this was done? Would be helpful to understand the rationale for allowing both of these scenarios.

Results:

1. The proportion of participants who were HIV positive is not shown in any table (including the supplemental ones); given the relevance of this population for TPT, this should be highlighted to understand its context in these geographies.
2. Given the variations in the strategies i.e., the entry point for TBD testing, it would be helpful to also show the Number-needed-to test (NNT) for each strategy. This would complement the costing perspective as well.

Discussion:

1. Interesting study and clearly presented.
2. The topic of TPT uptake is an important one and complex given the variation of approaches deployed across geographies and interpretation of WHO guidance. What is becoming clearer through studies like this one, is that simpler strategies (that rule of TBD) might be better.
3. In your study, your results showed no incident TB cases were reported in the No-TST arm compared to the other arms – does this make the point that better initial screening (with CXR) helps to ensure that no cases of TB were missed during contact investigation? Would suggest exploring this point further in the discussion.
4. Household contact investigation is a costly intervention, including the need for multiple visits by HHCs (to healthcare facilities) or healthcare staff to households. This is further compounded by TST testing (where multiple visits to read induration are required) – is there data available to show the number of household visits conducted to complete the TBI investigation, and if this influenced the health system or participant costs?
5. Gupta et al (Clinical Infectious Diseases; 2020) reported an interesting finding in a feasibility study conducted to inform the development of a TPT interventional trial where they assessed eligibility of HHCs of DR-TB index patients for TPT based on their classification per WHO guidance (TBI status, <5 years, or having HIV infection). They reported among 1007 HHCs, 775 (77%) were considered high-risk per these mutually exclusive groups. This highlighted the possibility that given the high proportion eligible, HHCs should be started on TPT by focusing on ruling on TB disease rather than performing other investigations, like TB infection testing. In essence, it made the case for simplifying the intervention in TB households. Importantly, these were DR-TB households, and the TB infection prevalence (72%) was quite high, and likely to be different in DS-TB households. It would be helpful to reflect on this study and its relevance given what you have found with your findings – does this support a similar approach? Does your HHC data look similar?
6. There are known challenges associated with implementing TST testing – was this observed in the study? Given the recent WHO-approval of TB specific skin tests, and their low costs (~$2), could this change the feasibility and costs associated with TB infection testing as implemented in your study?
7. PLHIV was noted in the introduction as the one high-risks groups (as per WHO) where UNHLM TPT targets were achieved – there is no specific discussion or data presented on this and its relevance in your study; what does this suggest for low HIV-prevalence settings?
