# Supplementary material for: Rapid molecular testing or chest X-ray or tuberculin skin testing for household contact assessment of tuberculosis infection: A cluster-randomized trial
Source: PLoS Med. 2025 Jul 28;22(7):e1004666. doi: 10.1371/journal.pmed.1004666 (PMC12316388; doi:10.1371/journal.pmed.1004666)
Supplement: Attachment — Submitted filename: ResponseToReviewer GXT PLOSMed R1.docx [file pmed.1004666.s009.docx]

To

Heather Van Epps, PhD, Executive Editor

And,

Syba Sunny, MBBS, MRes, FRCPath, Senior Editor

PLOS Medicine

Jan 15, 2025,

Dear Drs Van Epps and Sunny,

Many thanks for your email of Dec 10, 2024. We have addressed all questions and made many revisions in the text and tables. These changes, or in a few cases, reasons for not making requested changes, are detailed below. The revisions are also in red fint in the MARKED R1 versions, and we have also prepared CLEAN versions – easier to read perhaps.

We hope the revised manuscript is now acceptable for publication in PLOS Medicine, and look forward to hearing further from you

Menonli Adjobimey and Dick Menzies, on behalf of all authors

**Response to: Comments from the editors:**

1. Please ensure that your abstract is formatted according to CONSORT for Abstracts. Details are included in the general editorial points outlined below.

Response: We have checked and re-formatted.

2. Please add the dates of recruitment (month, day, year) to the abstract.

Response: dates were added. Line 49.

3. Our general rule for secondary outcomes in the abstract is that they should all be included or none should be included (ie, report only the primary outcome, per CONSORT). If you wish to include secondary outcomes in the abstract, you should report the results for each of them. Given the number of secondary outcomes included in the trial, our preference would be for your abstract to report only the primary outcome.

Response: We prefer to report all outcomes, as the primary outcome does not tell the whole story. Added to abstract (Lines 55-61)

4. Your current Data Availability statement requires additional detail and explanation. The PLOS Data Availability Policy is here: <http://journals.plos.org/plosmedicine/s/data-availability>). In brief, we require that all data underlying the study's findings be provided in a repository or as Supporting Information. For data residing with a third party, authors are required to provide instructions with contact information (web or email address) for obtaining the data. Please note that a study author cannot be the contact person for the data. Given that these are not third party data, we would require you to provide the minimal data set within the paper; ie, the data required to replicate the findings of a study, along with related metadata and methods. This includes the dataset used to reach the conclusions in the manuscript, any additional data needed to replicate the study findings, and related metadata and methods.

Response: We have prepared a data set. We would prefer to have this hosted by a public repository such as C-PATH, but have not yet initiated this process. We can send to you this data set, but what will you do with it? What control is there over access and use? At minimum assurances that the data is not used for commercial purposes or for profit, and that we are acknowledged?

5. Tables: Please use standard formatting for footnotes, removing labels (eg, abbreviations, notes).

Response: We have corrected the tables especially the footnotes, according to Plos Medicine instructions for authors.

------------------------------

-----------------------------

**Response to Comments from the reviewers:**

Reviewer #1: Statistical review

This trial reports a cluster randomised trial comparing different strategies for testing contacts of TB diagnosed individuals. Generally the trial appears to have been conducted well and provides interesting data. I have some comments on the statistical methods and reporting, provided below:

1. Abstract: I would recommend that the primary outcome proportion is provided for each group in addition to what is currently reported.

Response: This information has been added Line 54

2. Interventions: As a non-expert, I did not quite follow how the experimental arms would be expected to improve the TPT initiation rate (which would presumably be the case with a superiority trial). Could this be spelt out a bit more?

Response: An explanation has been added in the two paragraphs describing these interventions See Lines 151-153, and lines 158-159)

3. Methods: I would recommend an 'Outcomes' subsection is created where all the primary and secondary outcomes are listed. I note later that these are laid out in the analysis section, so perhaps an alternative is to add 'Outcomes' to that subsection title.

Response: An outcome subsection has been added to the Methods section (Lines 175-180)

4. Sample size calculation: I assume the power calculation used a two-sided 5% type I error rate: could this be added? Also, was clustering allowed for? Regardless, it would be useful to estimate the clustering effect and provide this for the benefit of future sample size calculations.

Response: Yes, a two-sided test was used; this has been specified (Line 247), as has the clustering (Lines 248-249). Clustering was accounted for (from the protocol “*The intra-class correlation coefficient (ICC) or clustering effect of households on LTBI treatment initiation was estimated from the ICC for completion in the adult trial comparing 4R with 9H [16], among study subjects who had at least one other family member in the study – i.e. from participants in families of size >1. We expect the average number of household contacts to be 3 (based on our just completed ACT4 study)*”.

As requested the cluster effect on TPT starting in this trial was estimated and has been added to the results (Lines 364-365).

5. Data analysis - secondary: it wasn't clear to me what analysis method was used for Incident TB Disease among HHC to allow for the variable follow-up.

Response: We used person time as specified in the analysis section (Lines 285-286)

6. Results - the sample size calculation is written as if the main outcome is the proportion of those randomised who initiated TPT, but the results are written as the proportion of those who were eligible. I see the registration page uses the latter, but isn't the former also a valid randomised comparison to do?

Response: The problem with using the proportion of those randomized who start TPT is that this will make the third strategy look vastly superior, but entirely because of protocol mandated management. In this study, in the no-TST strategy participants were ‘eligible for TPT’ as long as TB disease was excluded, meaning that over 95% were eligible (and TPT recommended) and so a very high proportion should have started TPT. On the other hand in the two other strategies which incorporated TB Infection testing, TPT was not recommended (per protocol – following WHO guidelines) for those who were TST (or IGRA) negative. Since more than half were TST negative – that meant that already half of all randomized were not recommended to start TPT. This is explained in Lines 292-299 (originally there, so not revised).

7. Table 4 - is there a way of getting uncertainty estimates for the differences in cost-effectiveness between arms? It would be interesting to know whether observed differences are beyond that consistent with variability. If this is possible, I would add this to the abstract too.

Response: Good idea. Although since the costs were directly measured (at least based on directly measured unit costs and number of health care activities) we are able to calculate 95% CI, rather than modeled uncertainty intervals. Has been added to Table 4, and also in Abstract (lines 59-61).

James Wason

Reviewer #2:

In this study, Adjobimey and colleagues conducted an RCT in Benin and Brazil to investigate three different strategies for managing household contacts of people with TB. Approaches included combinations of symptom screening, TST, chest x-ray, and rapid molecular testing. The primary outcome was initiation of TPT, which was found to be similar across the three arms. The inclusion of a costing analysis as part of the initial manuscript disseminating trial results is quite novel in TB, and is a relevant inclusion for a paper focused on, essentially, modes of preventing TB. This seems to be a well-conducted study with many findings of interest to the field. Some specific comments for consideration are below.

Major comments:

1. Methods, CXR reading: was there a pre-specified plan stating definitions of good or poor agreement between readers? How 'poor' would agreement need to be before an expert reader had to review all CXRs? Could a short explanation addressing this be included in this paragraph? - updated - From reading the results, seems a kappa-statistic was calculated (regular or weighted or other method, unsure) - I think this could be more clearly indicated in the methods section. Was agreement mainly influenced by a high proportion of 'normal' CXRs? Including positive percent agreement and negative percent agreement in results could clearly indicate where reviewers agreed. Is the number of CXRs in each category (abnormal-not TB/abnormal-possible TB/normal) ever stated in the paper? If not, can this be included in the paper/supplement somewhere?

Response: As the reviewer notes we did use kappa values to judge agreement, and used traditional thresholds for kappa to categorize agreement as poor, fair, good etc. But this was not specified in Methods and should have been, so has now been added (Lines 209-214). We have also added the number of CXR interpreted as normal/abnormal – overall and by strategy (in Results Lines 343-344).

2. Methods, Data analysis - Secondary: 3. Incident TB Disease: I am happy to be convinced that the described method is the best approach to estimating TB disease incidence rate. But I also wanted to check whether the author team had considered that the TBI risk may not be uniform across time, and therefore summarising measures per 100 person-years may not be the ideal approach? Would it perhaps be informative to also perform a survival analysis, stratify based on experimental strategy, and run a test to check for statistically-significant differences between curves/arms? This ultimately may not tell us much due to relatively low frequency of events, but assumedly since authors have follow-up times available it could be incorporated into analysis.

Response: For the prevalent TB we do not think time to event is relevant, since all were detected within 3 months of randomization (and are assumed to have been present at the time of randomization). For the incident TB, we agree that those may be interesting methods to explore but we are reluctant to test for significance when we initially stated we would NOT test but rather simply describe the TB incidence, since we expected the event rate to be low and there would be few incident TB events limiting power (as we found). As well, there seems to be a difference between strategies (see Table 5); hence we think that testing for significance now (ie post hoc) would violate the principles of statistical testing.

Minor comments:

3. Title: the title actually doesn't really mention TB/TBI - suggest modifying so non-TB people have a clearer idea of what the study is focussed on.

Response: Good point. We have added this.

4. Abstract, Background: it is stated that WHO recommends evaluation of all HHCs, but should clearly specify what the evaluation is actually for, i.e., the use-case. As stated, this paragraph is a little bit vague.

Response: We have revised this paragraph – see Lines 38-39

5. Abstract, Methods: I think it needs to be mentioned somewhere here that HHCs' index case had drug-susceptible TB (i.e., drug-resistant TB excluded).

Response: We agree; this has been added (Line 42)

6. Introduction, first paragraph: as 2024 WHO Global TB Report has now been published, would recommend updating figures to be consistent with new report.

Response: We have updated the statements (Lines 71-72), and the reference.

7. Introduction, third paragraph: author team writes that 'Provision of TPT to persons with undetected TB disease could result in acquired drug resistance' - but link to preceding sentences isn't fully clear. I think this is supposed to indicate that TPT is, unlike in the trial context, frequently given to people who have not had TBD excluded. Would strongly suggest making this more explicit in-text (with a citation), to strengthen the argument and relevance of the potential (and possibly common) harm that may arise from TPT.

Response: We agree that this sentence was out of place and have moved to the next paragraph, where exclusion of TB disease is reviewed. (now Line 97-99)

8. Introduction, fourth paragraph: suggest spelling out the interventions/experimental evaluations in each arm, as this is a statement of the study's main objective.

Response: We agree and have added a brief description of the strategies to the 4th paragraph. (Lines 107-108)

9. Methods, Study sites: further information about selected clinics would be helpful for understanding the 'kind' of index cases recruited, and in-turn, the risk their TB poses to their household contacts (which may be relevant when considering generalisability of findings). Could some detail about clinics be included in the supplement? Were these outpatient clinics at general hospitals? District level health centres? Outpatient clinics of respiratory hospitals? Something else? As well, does 'diagnostic facilities' refer to the diagnosis of TB disease or TB infection?

Response: We have added some description of the different sites to the text which ranged from primary care clinics to out-patient cinics in tertiary centres. This range of settings was deliberate as we felt that the results would be more generalizable to different settings in low-middle income countries. (lines 121-123)

10. Methods, Experimental 1 - rapid molecular tests: confirming whether the Xpert test used was Xpert MTB/RIF or Xpert MTB/RIF Ultra?

Response: Thank you for this observation, it was Ultra. This has been clarified in several places (Lines 124, 148, 156).

11. Methods, Data analysis - primary: Can you please state the primary outcome measure here? It's stated in abstract but not here really. As well, suggest rewording this paragraph to be a bit more specific and indicate that testing was for significant differences in *outcome* between arms, rather than 'significant differences between experimental arms'. When I first read this section, I did not understand what the outcome variable was - only when I read the results did I realise a risk difference was being calculated.

Response: We have added a subsection named outcomes (Lines 172-175), and amended the sentence in the analysis as suggested. (Lines 263-265)

12. Results, above Table 2: exactly what was the proportion of people initiating TPT in each arm? Since this is most related to the primary outcome, suggest describing these figures in-text somewhere.

Response: The proportions were added (Lines 354-357).

13. Table 2: I think this table contains all the relevant information, but it needs to be tuned. What exactly does the row header 'Completed' mean? On first glance, I thought it meant TPT was completed, but I think it must be referring to study procedures. Is there a way to demarcate the values used to calculate the primary analysis (i.e., 'proportion of HHC who started TPT within 3 months of randomization among all HHC contacts eligible for TPT'.)

Response: We added “investigation” in the lines of strategy completed. The subtitle indicates that the denominator of the proportion indicated in the lines of TPT initiation is eligible for TPT. Same for diagnosis (denominator is those who completed investigations). We added the denominator for TPT completion as well.

14. Discussion: very thorough and contextualises the results for both healthcare systems and patients. The explanations about how the trial staff made the intervention 'patient-centred' were particularly welcome.

Response: Thank you.

Reviewer #3:

The manuscript presents a well-conducted and relevant study addressing the complexities of TB preventive treatment (TPT) implementation across varying settings. The abstract requires no changes, but the introduction needs updated references and expanded reasoning on disparities in TPT target achievement among populations, particularly PLHIV versus other groups. Some methodological clarifications are needed, including the handling of drug resistance testing, HIV status confirmation, and intervention-specific details on chest X-ray (CXR) and microbiological methods. Results would benefit from inclusion of HIV-positive participant proportions and Number-Needed-to-Test (NNT) data for strategic comparisons. The discussion is strong but could delve deeper into the role of initial screening, costs of household contact investigation, and the implications of simplified strategies on health systems. Additionally, comparing findings with Gupta et al. (2020) could enhance insights on feasibility and scalability. Addressing challenges with TB infection testing and reflecting on WHO-approved TB-specific skin tests would further strengthen the manuscript's practical relevance.

Response: Thank you for your comments. Many of the points are covered in the detailed comments and responses below. To be clear, HHCs with known HIV infection were **excluded** from the study, due to the WHO guidance that these individuals should be placed on TPT as rapidly as possible, after excluding TB disease, with a different algorithm for their management from other HHCs (age 5 and older) in the WHO Handbook. Hence there were no HIV positive HHCs enrolled. We have clarified the assessment of drug resistance among index TB patients, and HHCs with disease.

In the discussion, we have not made as many changes as this reviewer suggests, in view of the positive assessments of the Discussion by the other reviewers. However we have added mention of Gupta’s study, and the new more specific tuberculins.

Specific comments:

Abstract: No changes suggested

Introduction:

1. Update reference to the Global TB report as the 2024 version is now available.

Response: Also mentioned by other reviewers. We have updated.

2. It would be helpful to expand the reasoning for WHY/HOW was the TPT targets achieved among PLHIV, but not for other groups?

Response: We believe this was the impact of PEPFAR programs, more than anything else. Provision of TPT became a major target for these programs after 2018, and substantial resources were poured into this, with the results that milions of PLHIV received TPT between 2018 and 2023. This, plus the relevant reference describing this PEPFAR initiative, has been added to the Introduction (Lines 78-82).

3. In paragraph 2, there is mention of WHO not requiring the use of TBI testing or CXR, but TPT started based on risk group classification - this sentence is misleading, as the guidance still requires ruling out TBD or at least not having clinical suspicion for TB.

Response: The sentence is correct, in that TBI testing and CXR are not required prior to starting TPT. However we have clarified that at least symptom screening should be done with further testing, such as CXR if there are symptoms suggestive of possible TB disease.

4. Use of PLHIV and PHIV - be consistent with abbreviations.

Response: Good point. Now consistently abbreviated as PLHIV.

Methods:

1. Within the definition of index patients, the abbreviation for GeneXpert is used i.e. GX; please spell this out first before using the abbreviation. Also, clarify if this was GeneXpert MTB/Rif or Ultra.

Response: As above, this has been spelled out, and we clarified that GeneXpert Mtb/RIF Ultra was used.

2. Among index patients, how was drug resistance ruled out among those diagnosed on smear or culture - was additional reflex testing done to rule out such patients as having additional drug resistance; please clarify.

Response: Both in Brazil and Benin sites, TB disease is now primarily diagnosed with rapid molecular testing – (GX Ultra), thus Rifampin resistance was routinely assessed, but testing was not performed for susceptibility of other first line TB drugs, unless Rifampin resistance was detected. In that situation, the index patient, and their HHCs, were not eligible. Clarified now: See Lines 126-129

3. Among those HHC with a known HIV status (they were not tested by the study), how was the status confirmed; this is limitation since some (with an unknown status) would have been tested while the 'other group' would have been largely self-reported.

Response: HHCs self-reported HIV status, with those reporting a prior positive test were excluded. A handful of HHCs with unknown status were tested as their index TB patient was HIV positive (following WHO, and national guidelines). We have acknowledged this is a potential limitation in the Discussion (Lines 479-481).

4. As described within the 'Standard' intervention, was CXR read once (and a decision made) or were multiple reads conducted; it is assumed that this was done by human-readers and not aided by computer-aided detection software, however this should be explicitly stated. In addition, please clarify what microbiological tests were done.

Response: The CXR was read at study sites by site investigators/other providers and all management decisions (further testing/treatment) were based on that interpretation. This has been clarified in the text (Lines 205-206). We have added what microbiologic tests were done. Both in Benin and Brazil, this was a GX Ultra on a sputum sample. This information has been added to the text (Lines 148-149 and 153-55)

5. Was the same TPT used in all interventions? Currently this is described within the 'Experiment 2' paragraph, which suggests it only applied to this intervention; might help to have this separated if applied to all interventions.

Response: Thanks for pointing this out. We have moved the description of the TPT regimens to a separate section – so it does not seem to be limited to one strategy (Lines 172-174).

6. In the data analysis, treatment initiation was defined as receiving a prescription, or dispensed medications for TPT - these are two quite different prospects in relation to 'initiating treatment' i.e., pills in hand are close to initiation treatment versus having to still collect medication; is there a reason why this was done? Would be helpful to understand the rationale for allowing both of these scenarios.

Response: This reflects the realities of the TB programmes in the two different countries. In Benin, HHCs did not receive a prescription – but instead were given the TPT medications in the clinics where they were seen. In Brazil, in some clinics this was also the procedure followed, but in others the HHCs were given a prescription which they filled at the pharmacy, in the same clinic or the district pharmacy. We agree these are different phenomena, but since we also measured, and reported completion, this would help account for different probabilities of HHCs actually receiving TPT medications. We have added this as a potential limitation in the Discussion (Lines 481-486)

Results:

1. The proportion of participants who were HIV positive is not shown in any table (including the supplemental ones); given the relevance of this population for TPT, this should be highlighted to understand its context in these geographies.

Response: To reiterate – HHCs who were HIV positive were excluded from this study. We could not gather information on HIV status among index TB patients, (only age and microbiological information as this guided contact investigation) due to ethical restrictions, as we did not obtain consent form index patients – only their verbal agreement to allow us to contact their HHC’s. (see Supplemental Table 1)

2. Given the variations in the strategies i.e., the entry point for TBD testing, it would be helpful to also show the Number-needed-to test (NNT) for each strategy. This would complement the costing perspective as well.

Response: It is unclear to us how we would calculate this – since the testing strategies are, by design, so different. And what endpoint is being suggested? If the endpoint is Starting TPT, or completing investigations – we have shown no significant difference between strategies. So comparing NNT does not seem useful. As well, we cannot compare NNT for TB infection testing (ie TST) since the 3rd strategy explicitly skipped that testing. Similarly, if we wanted to compare number needed to perform CXR in order to start TPT – that would also be impossible, since one strategy did not include CXR. Bottom line, we have not added this analysis.

Discussion:

1. Interesting study and clearly presented.

Response: Thank you

2. The topic of TPT uptake is an important one and complex given the variation of approaches deployed across geographies and interpretation of WHO guidance. What is becoming clearer through studies like this one, is that simpler strategies (that rule of TBD) might be better.

Response: It is interesting to us that this Reviewer draws a different conclusion from our findings than we do. We found no difference in the proportion ***of eligible*** HHCs who started TPT between all 3 strategies – two of which included TB Infection testing and one did not. We also found that TPT completion was better in the “more complex” strategies (with TBI testing). Our conclusion is that the more complex strategies are as readily completed as the simpler one, with the Advantage of better TPT completion. Hence, we feel our findings support the more complex investigations (basically adding TB infection testing) as this identified persons at higher risk of disease, and prevents those at lower risk from being exposed to potentially harmful TPT (admittedly these benefits were not shown in our study, but are based on extensive published evidence). In light of the reviewer’s comments, we have gone over the Discussion again to clarify our take on our findings! (see Lines 487-490 and 507-509).

3. In your study, your results showed no incident TB cases were reported in the No-TST arm compared to the other arms - does this make the point that better initial screening (with CXR) helps to ensure that no cases of TB were missed during contact investigation? Would suggest exploring this point further in the discussion.

Response: Again, we point out that we expected low numbers of persons with disease, and so did not plan testing for statistical significance. The difference is interesting, but we feel this may be due to chance, and so feel we must be VERY cautious in discussing these findings. We have explored this finding briefly (Lines 510-514).

4. Household contact investigation is a costly intervention, including the need for multiple visits by HHCs (to healthcare facilities) or healthcare staff to households. This is further compounded by TST testing (where multiple visits to read induration are required) - is there data available to show the number of household visits conducted to complete the TBI investigation, and if this influenced the health system or participant costs?

Response: This is an interesting point. Home visits were made in Benin, where in a prior study these were found to be essential for adequate HHC investigation. Hence this has been adopted as standard care by the TB programme there. Home visits are used to identify contacts and refer them to clinical facilities for investigation. To expedite investigations HHCs underwent symptom screening during these home visits. In Brazil home visits were rarely done for HHC management, although in many sites community health workers (who have low wages) routinely visit to follow the index patients.

It is important to point out that in both countries the clinical staff coordinated investigations to complete as many as possible at the same time (eg TST reading and CXR or GX-Ultra during the same visit if TST positive). This is shown in the Supplemental Figures summarizing the strategies. This so-called “one stop shopping” helped to expedite investigations while reducing time and cost burden on participating HHCs. We had already discussed this point in the Discussion, so have left it unchanged (Lines 491-494)

5. Gupta et al (Clinical Infectious Diseases; 2020) reported an interesting finding in a feasibility study conducted to inform the development of a TPT interventional trial where they assessed eligibility of HHCs of DR-TB index patients for TPT based on their classification per WHO guidance (TBI status, <5 years, or having HIV infection). They reported among 1007 HHCs, 775 (77%) were considered high-risk per these mutually exclusive groups. This highlighted the possibility that given the high proportion eligible, HHCs should be started on TPT by focusing on ruling on TB disease rather than performing other investigations, like TB infection testing. In essence, it made the case for simplifying the intervention in TB households. Importantly, these were DR-TB households, and the TB infection prevalence (72%) was quite high, and likely to be different in DS-TB households. It would be helpful to reflect on this study and its relevance given what you have found with your findings

- does this support a similar approach? Does your HHC data look similar?

Response: There are some very major differences between our study and the study by Gupta et al – the design, and most importantly the index patients. Gupta’s cohort study, was of HHCs whose index patients had MDR/RR-TB – leading to longer and likely more intense exposure than our patient population, with the result that prevalence of positive TST was much higher than in our study. We have mentioned this study and pointed out these important differences. (Lines 521-526)

.

6. There are known challenges associated with implementing TST testing - was this observed in the study? Given the recent WHO-approval of TB specific skin tests, and their low costs (~$2), could this change the feasibility and costs associated with TB infection testing as implemented in your study?

Response: Access to tuberculin testing material with stock-outs has been an issue in these countries, reflecting global shortages of PPD. We agree the new tuberculins offer substantial promise especially at current prices of $1.50 per dose if purchased through the Global Drug Facility. In the study, we procured tuberculin for both countries so this was not an issue per se, but we agree this problem is important, and new TBST offer a promising solution (see Discussion Lines 494-497)

7. PLHIV was noted in the introduction as the one high-risks groups (as per WHO) where UNHLM TPT targets were achieved - there is no specific discussion or data presented on this and its relevance in your study; what does this suggest for low HIV-prevalence settings?

Response: We agree that TPT coverage for PLHIV has increased very substantially in the last 10 years. We think this is largely the result of PEPFAR programs, with allocation of substantial resources To explain this we have added details (and a reference) in the Introduction. (See Lines 78-82).

Any attachments provided with reviews can be seen via the following link: <https://www.editorialmanager.com/pmedicine/l.asp?i=960323&l=XB710QD0>

--------------------------------------------------------- --- General editorial requests:

(Note: not all will apply to your paper, but please check each item carefully)

*	We ask every co-author listed on the manuscript to fill in a contributing author statement, making sure to declare all competing interests. If any of the co-authors have not filled in the statement, we will remind them to do so when the paper is revised. If all statements are not completed in a timely fashion this could hold up the re-review process. If new competing interests are declared later in the revision process, this may also hold up the submission. Should there be a problem getting one of your co-authors to fill in a statement we will be in contact. Please do not add or remove authors without first discussing this with the handling editor. You can see our competing interests policy here: <http://journals.plos.org/plosmedicine/s/competing-interests>.

*	Please upload any figures associated with your paper as individual TIF or EPS files with 300dpi resolution at resubmission; please read our figure guidelines for more information on our requirements: <http://journals.plos.org/plosmedicine/s/figures>. While revising your submission, please upload your figure files to the PACE digital diagnostic tool, <https://pacev2.apexcovantage.com/>. PACE helps ensure that figures meet PLOS requirements. To use PACE, you must first register as a user. Then, login and navigate to the UPLOAD tab, where you will find detailed instructions on how to use the tool. If you encounter any issues or have any questions when using PACE, please email us at PLOSMedicine@plos.org.

*	As above, please ensure that the paper adheres to the PLOS Data Availability Policy (see <http://journals.plos.org/plosmedicine/s/data-availability>), which requires that all data underlying the study's findings be provided in a repository or as Supporting Information. For data residing with a third party, authors are required to provide instructions with contact information (web or email address) for obtaining the data. Please note that a study author cannot be the contact person for the data. PLOS journals do not allow statements supported by "data not shown" or "unpublished results." For such statements, authors must provide supporting data or cite public sources that include it.

*	We expect all researchers with submissions to PLOS in which author-generated code underpins the findings in the manuscript to make all author-generated code available without restrictions upon publication of the work. In cases where code is central to the manuscript, we may require the code to be made available as a condition of publication. Authors are responsible for ensuring that the code is reusable and well documented. Please make any custom code available, either as part of your data deposition or as a supplementary file. Please add a sentence to your data availability statement regarding any code used in the study, e.g. "The code used in the analysis is available from Github [URL] and archived in Zenodo [DOI link]" Please review our guidelines at <https://journals.plos.org/plosmedicine/s/materials-software-and-code-sharing> and ensure that your code is shared in a way that follows best practice and facilitates reproducibility and reuse. Because Github depositions can

be readily changed or deleted, we encourage you to make a permanent DOI'd copy (e.g. in Zenodo) and provide the URL.

*	Please ensure that the study is reported according to the CONSORT for cluster randomized trials guideline and include the completed CONSORT checklist as Supporting Information (<https://www.equator-network.org/reporting-guidelines/consort-cluster/>). When completing the checklist, please use section and paragraph numbers, rather than page numbers. Please add the following statement, or similar, to the Methods: "This study is reported as per the CONSORT for cluster randomized trials guideline (S1 Checklist)."

FORMATTING - GENERAL

*	Abstract: Please structure your abstract using the PLOS Medicine headings (Background, Methods and Findings, Conclusions). Please combine the Methods and Findings sections into one section.

*	At this stage, we ask that you include a short, non-technical Author Summary of your research to make findings accessible to a wide audience that includes both scientists and non-scientists. The Author Summary should immediately follow the Abstract in your revised manuscript. This text is subject to editorial change and should be distinct from the scientific abstract. Ideally each sub-heading should contain 2-3 single sentence, concise bullet points containing the most salient points from your study. In the final bullet point of 'What Do These Findings Mean?', please include the main limitations of the study in non-technical language. Please see our author guidelines for more information: <https://journals.plos.org/plosmedicine/s/revising-your-manuscript#loc-author-summary>.

*	Please express the main results with 95% CIs as well as p values. When reporting p values please report as p<0.001 and where higher as the exact p value p=0.002, for example. Throughout, suggest reporting statistical information as follows to improve clarity for the reader "22% (95% CI [13%,28%]; p</=)". Please be sure to define all numerical values at first use.

*	Please include page numbers and line numbers in the manuscript file. Use continuous line numbers (do not restart the numbering on each page).

*	Please cite the reference numbers in square brackets. Citations should precede punctuation.

FIGURES AND TABLES

*	Please provide titles and legends for all figures and tables (including those in Supporting Information files).

*	Please define all abbreviations used in each figure/table (including those in Supporting Information files).

*	Please consider avoiding the use of red and green in order to make your figure more accessible to those with color blindness.

SUPPLEMENTARY MATERIAL

*	Please note that supplementary material will be posted as supplied by the authors. Therefore, please amend it according to the relevant comments outlined here.

*	Please cite your Supporting Information as outlined here: <https://journals.plos.org/plosmedicine/s/supporting-information>

REFERENCES

*	PLOS uses the numbered citation (citation-sequence) method and first six authors, et al.

*	Please ensure that journal name abbreviations match those found in the National Center for Biotechnology Information (NCBI) databases (<http://www.ncbi.nlm.nih.gov/nlmcatalog/journals>), and are appropriately formatted and capitalised.

*	Where website addresses are cited, please include the complete URL and specify the date of access (e.g. [accessed: 12/06/2023]).

*	Please also see <https://journals.plos.org/plosmedicine/s/submission-guidelines#loc-references> for further details on reference formatting.

Randomized controlled trials

*	Please structure the Methods section using the following sub-headings: Study design and participants, Randomization and masking, Procedures, Outcomes, Statistical analysis.

*	Please ensure that the outcomes in the submitted manuscript match those outlined the protocol [and/or trial registry]. Please clarify and explain all discrepancies between the paper and protocol. If the outcomes were not prespecified in the protocol, please define them in the Methods (Outcomes section) as post hoc and explain why they were added. Post-hoc comparisons should be presented as hypothesis generating rather than conclusive.

*	Please ensure that all prespecified outcomes (primary, secondary, and exploratory) are listed in the Methods/Outcomes section and indicate whether there are outcomes that are not presented in the current report.

*	Please specify the dates (Month, Day, Year) during which study enrollment and follow up occurred.

*	Please include absolute numbers wherever you report percentages; eg, n/N (%)

*	Please complete the CONSORT checklist (<https://www.equator-network.org/reporting-guidelines/consort/>) and ensure that all components of CONSORT are present in the manuscript, including how randomization was performed, allocation concealment, blinding of intervention, definition of lost to follow-up, power statement. When completing the checklist, please use section and paragraph numbers, rather than page numbers.

*	Please report your abstract according to CONSORT for abstracts, following the PLOS Medicine abstract structure (Background, Methods and Findings, Conclusions) <https://www.equator-network.org/reporting-guidelines/consort-abstracts/>

*	If your trial had to undergo important modifications in response to extenuating circumstances, please complete the CONSERVE-CONSORT checklist and provide in your Supporting Information; (<https://www.equator-network.org/reporting-guidelines/guidelines-for-reporting-trial-protocols-and-completed-trials-modified-due-to-the-covid-19-pandemic-and-other-extenuating-circumstances-the-conserve-2021-statement/>). When completing the checklist, please use section and paragraph numbers, rather than page numbers.

*	In keeping with our commitment to Open Science, please include the study protocol document and analysis plan (including any amendments) as Supporting Information to be published with the manuscript if accepted.

*	Please note that PLOS Medicine requires prospective, public registration of a data sharing plan (as part of mandatory clinical trials registration) for all clinical trials that began enrollment on or after January 1, 2019, in accordance with ICMJE requirements.
