# Supplementary material for: Rapid molecular testing or chest X-ray or tuberculin skin testing for household contact assessment of tuberculosis infection: A cluster-randomized trial
Source: PLoS Med. 2025 Jul 28;22(7):e1004666. doi: 10.1371/journal.pmed.1004666 (PMC12316388; doi:10.1371/journal.pmed.1004666)
Supplement: Attachment — Submitted filename: GXT ResponseToReviewers R3.docx [file pmed.1004666.s010.docx]

**June 17, 2025**

**RESPONSE (FIRST):**

Dear Dr Farrell,

Apologies for the near-3 month delay. I did not realize this process took so long

I now have a data transfer agreement signed, and an account, and now Vivli has assigned a study DOI number, which I have also added in the paper (Lines 573-575)

I think that takes care of the last outstanding item

Let me know if the paper is now fully accepted for PLOS Med

Many thanks for your patience

Dick Menzies

**PS – I AM ONLY UPLOADING THREE NEW FILES – this response, and the R3 version (clean and marked). All other files remain the same (Figure, Supplemental files)**

**Here is the letter from VIVLI – public data repository:**

**From:** Sarah Sweet (Vivli User Support) <support@vivli.org>
**Sent:** Monday, June 16, 2025 4:16 PM
**To:** Richard Ian Menzies, Dr <dick.menzies@mcgill.ca>
**Subject:** [Vivli] Re: RE: Your last email to me Action Required for Vivli Single Study Submission N/A

**Sarah Sweet** (Vivli)

Jun 16, 2025, 16:16 EDT

Dear Dr. Menzies,
 
Your study has now been posted. Here is your study DOI that you may want to use in your publication to direct researchers to where they can access the data underlying -- [https://doi.org/10.25934/PR00011500](https://can01.safelinks.protection.outlook.com/?url=https%3A%2F%2Fdoi.org%2F10.25934%2FPR00011500&data=05%7C02%7Cdick.menzies%40mcgill.ca%7C8129da088b1b4dfc73b108ddad12a6fc%7Ccd31967152e74a68afa9fcf8f89f09ea%7C0%7C0%7C638857018450082329%7CUnknown%7CTWFpbGZsb3d8eyJFbXB0eU1hcGkiOnRydWUsIlYiOiIwLjAuMDAwMCIsIlAiOiJXaW4zMiIsIkFOIjoiTWFpbCIsIldUIjoyfQ%3D%3D%7C0%7C%7C%7C&sdata=oWw0skSkIiA3R%2BNC0aLe7ssmLxf5Zc1bBaC7VKEPJ%2Bo%3D&reserved=0)
 
Using publicly available information and what you have provided to us, we have included key metadata elements. Of course, you can always send us additional information or updates for inclusion to add to the metadata about your study.
 
As a next step, please have the member of your team will be uploading the data, create a Vivli account. Detailed guidance on creating an account can be found here:
[https://vivli.org/Vivli_quick_start_users_training](https://can01.safelinks.protection.outlook.com/?url=https%3A%2F%2Fvivli.org%2FVivli_quick_start_users_training&data=05%7C02%7Cdick.menzies%40mcgill.ca%7C8129da088b1b4dfc73b108ddad12a6fc%7Ccd31967152e74a68afa9fcf8f89f09ea%7C0%7C0%7C638857018450102323%7CUnknown%7CTWFpbGZsb3d8eyJFbXB0eU1hcGkiOnRydWUsIlYiOiIwLjAuMDAwMCIsIlAiOiJXaW4zMiIsIkFOIjoiTWFpbCIsIldUIjoyfQ%3D%3D%7C0%7C%7C%7C&sdata=4PzAXOxgoGclYpQNFjPhdADrELv9vjJGWzUsPAFN3m8%3D&reserved=0)
 
Once completed, please send their contact information so we can invite them to join your organization and grant access for data upload.
 
We would also like to arrange the invoicing for your study listing. Please see the following data sharing page, "Researcher Data Sharing Costs" tab, for information on data sharing costs and confirm the size of your dataset: [https://vivli.org/resources/sharedata/,](https://can01.safelinks.protection.outlook.com/?url=https%3A%2F%2Fvivli.org%2Fresources%2Fsharedata%2F%2C&data=05%7C02%7Cdick.menzies%40mcgill.ca%7C8129da088b1b4dfc73b108ddad12a6fc%7Ccd31967152e74a68afa9fcf8f89f09ea%7C0%7C0%7C638857018450129018%7CUnknown%7CTWFpbGZsb3d8eyJFbXB0eU1hcGkiOnRydWUsIlYiOiIwLjAuMDAwMCIsIlAiOiJXaW4zMiIsIkFOIjoiTWFpbCIsIldUIjoyfQ%3D%3D%7C0%7C%7C%7C&sdata=XtHXCj%2Bpg8r6p9yUs29TQN%2BfGqy%2FrwzYvzM1Hmalf2o%3D&reserved=0) please also confirm the correct invoicing contact you have provided in your study submission (dick.menzies@mcgill.ca) - we will then prepare and send the invoice accordingly.
 
If you have any questions, please do not hesitate to let ask.
 
Warm regards,
The Vivli Team

**EARLIER RELATED CORRESPONDENCE**

**From:** Alison Farrell <afarrell@plos.org>
**Sent:** Friday, June 13, 2025 2:27 PM
**To:** Richard Ian Menzies, Dr <dick.menzies@mcgill.ca>
**Subject:** RE: PLOS MED PMEDICINE-D-24-03440R2 Decision and Vivli Single Study Submission with ID: NCT04528823 has been accepted for processing by Vivli

Yes, please do. Publication will likely be delayed until we get confirmation that the data can be accessed, but we should be able to start the process.

Thanks,

Alison

**From:** Richard Ian Menzies, Dr <[dick.menzies@mcgill.ca](mailto:dick.menzies@mcgill.ca)>
**Sent:** Friday, June 13, 2025 9:24 AM
**To:** Alison Farrell <[afarrell@plos.org](mailto:afarrell@plos.org)>
**Subject:** RE: PLOS MED PMEDICINE-D-24-03440R2 Decision and Vivli Single Study Submission with ID: NCT04528823 has been accepted for processing by Vivli

OK

So, should we officially re-submit?

How to proceed?

Thanks for letting me know

Dm,

**From:** Alison Farrell <[afarrell@plos.org](mailto:afarrell@plos.org)>
**Sent:** Friday, June 13, 2025 10:53 AM
**To:** Richard Ian Menzies, Dr <[dick.menzies@mcgill.ca](mailto:dick.menzies@mcgill.ca)>
**Subject:** RE: PLOS MED PMEDICINE-D-24-03440R2 Decision and Vivli Single Study Submission with ID: NCT04528823 has been accepted for processing by Vivli

Yes! I think this works, thanks!

Alison

**From:** Richard Ian Menzies, Dr <[dick.menzies@mcgill.ca](mailto:dick.menzies@mcgill.ca)>
**Sent:** Friday, June 13, 2025 7:49 AM
**To:** Alison Farrell <[afarrell@plos.org](mailto:afarrell@plos.org)>
**Subject:** FW: PLOS MED PMEDICINE-D-24-03440R2 Decision and Vivli Single Study Submission with ID: NCT04528823 has been accepted for processing by Vivli

Hi again

I just got this today

Is this enough to proceed

Data not actually transferred

But as you can see we are making progress…

dm

**From:** Vivli Center <[noreply@vivli.org](mailto:noreply@vivli.org)>
**Sent:** Friday, June 13, 2025 9:31 AM
**To:** [info@vivli.org](mailto:info@vivli.org)
**Subject:** Vivli Single Study Submission with ID: NCT04528823 has been accepted for processing by Vivli

The Vivli Single Study Submission with ID: NCT04528823 from Research Institute of the McGill University Health Centre has been accepted and Vivli will begin preparing this study for posting

[Click here](https://can01.safelinks.protection.outlook.com/?url=http%3A%2F%2Furl970.vivli.org%2Fls%2Fclick%3Fupn%3Du001.9QEtwtufxLhnbWZ1-2FpSbt8O005aNB5slppyKhr0xXJhcG1COT2vAwcIh-2BFt0BRKMQfyYTHJBKDERBR4ffQEgT1LgIv9ruMTLhBsGGwH4OjP-2B-2FhkU-2FGFl-2Bdg-2BsCo0mbKwNxcg_EqxZANw1qm1VO5MCgb-2FDHetZ2u-2B6gGOEp7jiyhLFqwJBrbks5UxAbgK97jlnaECK38g8NIem1rOFIHOJY29XkLooFeFyP2vW6i4dXPJoSxn06ToSSRCkiO5xZZn5iKeklSgvL6G4DZcG0EIgoJhmg2SQSd5QZ4kcSn386FCR-2Fg85Z2rQwrH0lwQzp-2FEqF9y9gulW-2BU37q6MRKiiS6J-2FKSg-3D-3D&data=05%7C02%7Cdick.menzies%40mcgill.ca%7Cacd96f7d6b8941e6eef708ddaaa7ed6e%7Ccd31967152e74a68afa9fcf8f89f09ea%7C0%7C0%7C638854360455296047%7CUnknown%7CTWFpbGZsb3d8eyJFbXB0eU1hcGkiOnRydWUsIlYiOiIwLjAuMDAwMCIsIlAiOiJXaW4zMiIsIkFOIjoiTWFpbCIsIldUIjoyfQ%3D%3D%7C0%7C%7C%7C&sdata=e7MIeXvWuIJK6lvt7PJf8TWcVMwC8w97Bbbs5%2BCkbjk%3D&reserved=0) to access it

Thanks,
The Vivli Team

**EARLIER CONDITIONAL ACCEPTANCE LETTER FROM YOU**

March 20 2025

Dear Dr. Menzies,

Thank you very much for re-submitting your manuscript "Rapid molecular testing or chest-X-ray or tuberculin skin testing for household contact assessment of tuberculosis infection: Results of a cluster-randomized trial" (PMEDICINE-D-24-03440R2) for review by PLOS Medicine.

I have discussed the paper with my colleagues and the academic editor and it was also seen again by three reviewers. I am pleased to say that provided the remaining editorial and production issues are dealt with we are planning to accept the paper for publication in the journal.

The remaining issues that need to be addressed are listed at the end of this email. Any accompanying reviewer attachments can be seen via the link below. Please take these into account before resubmitting your manuscript:

Please note that the data availability issue needs to be sorted prior to acceptance.

***Please note while forming your response, if your article is accepted, you may have the opportunity to make the peer review history publicly available. The record will include editor decision letters (with reviews) and your responses to reviewer comments. If eligible, we will contact you to opt in or out.***

In revising the manuscript for further consideration here, please ensure you address the specific points made by each reviewer and the editors. In your rebuttal letter you should indicate your response to the reviewers' and editors' comments and the changes you have made in the manuscript. Please submit a clean version of the paper as the main article file. A version with changes marked must also be uploaded as a marked up manuscript file.

Please also check the guidelines for revised papers at for any that apply to your paper.

If you haven't already, we ask that you provide a short, non-technical Author Summary of your research to make findings accessible to a wide audience that includes both scientists and non-scientists. The Author Summary should immediately follow the Abstract in your revised manuscript. This text is subject to editorial change and should be distinct from the scientific abstract. Author Summary added (254 words)

In addition to these revisions, you will need to complete some formatting changes, which you will receive in a follow up email. A member of our team will be in touch with a set of requests shortly.

We expect to receive your revised manuscript within 1 week. Please email us ([plosmedicine@plos.org](mailto:plosmedicine@plos.org)) if you have any questions or concerns.

We ask every co-author listed on the manuscript to fill in a contributing author statement. If any of the co-authors have not filled in the statement, we will remind them to do so when the paper is revised. If all statements are not completed in a timely fashion this could hold up the re-review process. Should there be a problem getting one of your co-authors to fill in a statement we will be in contact. YOU MUST NOT ADD OR REMOVE AUTHORS UNLESS YOU HAVE ALERTED THE EDITOR HANDLING THE MANUSCRIPT TO THE CHANGE AND THEY SPECIFICALLY HAVE AGREED TO IT.

Please ensure that the paper adheres to the PLOS Data Availability Policy (see <https://can01.safelinks.protection.outlook.com/?url=http%3A%2F%2Fjournals.plos.org%2Fplosmedicine%2Fs%2Fdata-availability&data=05%7C02%7Cdick.menzies%40mcgill.ca%7Cc1bc392913ff4f050ce708dd67fdd8cb%7Ccd31967152e74a68afa9fcf8f89f09ea%7C0%7C0%7C638781062175870829%7CUnknown%7CTWFpbGZsb3d8eyJFbXB0eU1hcGkiOnRydWUsIlYiOiIwLjAuMDAwMCIsIlAiOiJXaW4zMiIsIkFOIjoiTWFpbCIsIldUIjoyfQ%3D%3D%7C0%7C%7C%7C&sdata=%2FfDbEG2ba9ki%2FCXXrXK1rj9Fne1AcMtib5LjDIzyj6E%3D&reserved=0>), which requires that all data underlying the study's findings be provided in a repository or as Supporting Information. For data residing with a third party, authors are required to provide instructions with contact information for obtaining the data. PLOS journals do not allow statements supported by "data not shown" or "unpublished results." For such statements, authors must provide supporting data or cite public sources that include it.

To enhance the reproducibility of your results, we recommend that you deposit your laboratory protocols in protocols.io, where a protocol can be assigned its own identifier (DOI) such that it can be cited independently in the future. Additionally, PLOS ONE offers an option to publish peer-reviewed clinical study protocols. We have published the study protocol already. It is referenced in this paper

Please review your reference list to ensure that it is complete and correct. If you have cited papers that have been retracted, please include the rationale for doing so in the manuscript text, or remove these references and replace them with relevant current references. Any changes to the reference list should be mentioned in the rebuttal letter that accompanies your revised manuscript. None

Please note, when your manuscript is accepted, an uncorrected proof of your manuscript will be published online ahead of the final version, unless you've already opted out via the online submission form. If, for any reason, you do not want an earlier version of your manuscript published online or are unsure if you have already indicated as such, please let the journal staff know immediately at [plosmedicine@plos.org](mailto:plosmedicine@plos.org).

If you have any questions in the meantime, please contact me or the journal staff on [plosmedicine@plos.org](mailto:plosmedicine@plos.org).

We look forward to receiving the revised manuscript by Mar 27 2025 11:59PM.

Sincerely,

Alison Farrell, PhD

Senior Editor

PLOS Medicine

plosmedicine.org

------------------------------------------------------------

Requests from Editors:

Title:

Please revise according to PLOS Medicine format. “Results” should not be in the title.

E,g, Comparison of rapid molecular testing, chest-X-ray and tuberculin skin testing for household contact assessment of tuberculosis infection: a cluster-randomized trial. OK deleted

Abstract:

The Abstract consists of the following subheadings: Background, Methods and findings, Conclusions Please use the active voice throughout, including in the Abstract. E.g. We conducted a superiority, open-label, cluster-randomized trial…. Changed

Please clarify what is meant by “completion of investigations” as a secondary outcome. Please revise. Added details Please specify the secondary outcomes as per the protocol and report on the findings for all. All outcomes specified have been reported

Please clarify “Of 848 eligible” (add HHC). Added

Please remove the term ‘high-quality’ from the Abstract. Deleted and added ‘randomized’.

Please include a sentence on study limitations. OK added in the conclusions.

Please clarify whether, in the penultimate sentence of the abstract, you mean by “TBI testing” tuberculin skin testing/standard testing? Clarified (changed)

Please check compound adjectives, e.g. resource-limited? protocol-mandated? throughout the manuscript. Not sure what the issue is here??

Introduction:

TPT is defined as preventive TB treatment. Should this be TB preventive treatment? YES – sorry – have corrected.

Also, TPT should be explained (i.e. the general reader is unaware that it is a monotherapy until the last paragraph of the Intro). OK added a sentence

Please break penultimate sentence of first paragraph in two (presently too long), Two sentences now. Monotherapy should not be hyphenated. Corrected

In the last sentence of the Intro, please restate all three strategies, not simply one. Really, all three were there, but now clarified.

Results:

Please use active voice and add an introductory sentence to the first paragraph. We have changed to an active voice where it seemed most appropriate. We have not added an introductory sentence as we are unclear what you mean by this.

Omit repeat of “in Benin” in the 2nd sentence. Deleted.

Please state the numbers in each study arm in the first paragraph. Added

“Of those eligible, more than 95% started TPT”—please state the eligibility criteria. Added

Please add subheading to the Results. OK

Discussion:

HHCs not HHC’s corrected

Please avoid jargon, e.g. stock-out word changed

Please discuss the study limitations in the Discussion section. This is done in Paragraph 3 (15 lines devoted to limitations compared to 5 lines about strengths) – I have not added to this

We also request that you address the following PLOS Medicine requirements:

* In the last sentence of the Abstract Methods and Findings section, please describe the main limitation(s) of the study's methodology. As above – added a sentence

* PLOS Medicine requires that the de-identified data underlying the specific results in a published article be made available, without restrictions on access, in a public repository or as Supporting Information at the time of article publication, provided it is legal and ethical to do so. Please see the policy at

<https://can01.safelinks.protection.outlook.com/?url=http%3A%2F%2Fjournals.plos.org%2Fplosmedicine%2Fs%2Fdata-availability&data=05%7C02%7Cdick.menzies%40mcgill.ca%7Cc1bc392913ff4f050ce708dd67fdd8cb%7Ccd31967152e74a68afa9fcf8f89f09ea%7C0%7C0%7C638781062175903171%7CUnknown%7CTWFpbGZsb3d8eyJFbXB0eU1hcGkiOnRydWUsIlYiOiIwLjAuMDAwMCIsIlAiOiJXaW4zMiIsIkFOIjoiTWFpbCIsIldUIjoyfQ%3D%3D%7C0%7C%7C%7C&sdata=h6BlnW%2BKfzQUjmSIR%2F9snDMr8I%2FML3yWfk%2FTpLzLAC4%3D&reserved=0>

and FAQs at

<https://can01.safelinks.protection.outlook.com/?url=http%3A%2F%2Fjournals.plos.org%2Fplosmedicine%2Fs%2Fdata-availability%23loc-faqs-for-data-policy&data=05%7C02%7Cdick.menzies%40mcgill.ca%7Cc1bc392913ff4f050ce708dd67fdd8cb%7Ccd31967152e74a68afa9fcf8f89f09ea%7C0%7C0%7C638781062175924159%7CUnknown%7CTWFpbGZsb3d8eyJFbXB0eU1hcGkiOnRydWUsIlYiOiIwLjAuMDAwMCIsIlAiOiJXaW4zMiIsIkFOIjoiTWFpbCIsIldUIjoyfQ%3D%3D%7C0%7C%7C%7C&sdata=PCf0MkvGEA2zzEoS7CxXrOO4aQpBL1omN%2BWAKFwx5s0%3D&reserved=0>

* The Data Availability Statement (DAS) requires revision. For each data source used in your study:

a) If the data are freely or publicly available, note this and state the location of the data: within the paper, in Supporting Information files, or in a public repository, and include the DOI or accession number.

b) If the data are owned by a third party but freely available upon request, please note this and state the owner of the data set and contact information for data requests (web or email address). Note that a study author cannot be the contact person for the data.

c) If the data are not freely available, please describe briefly the ethical, legal, or contractual restriction that prevents you from sharing it. Please also include an appropriate contact (web or email address) for inquiries (again, this cannot be a study author).

* For studies in which a novel model is central to the manuscript's findings, if this is the case here, authors are responsible for providing the source code needed to replicate the study's findings in a repository (such as GitHub, SourceForge or Bitbucket) or a cloud computing service (such as Code Ocean). Protection of authors’ intellectual property will not be cause for exception. Please explain in the manuscript’s Data Availability Statement how readers can access the shared code if new code was generated.

**Responses to Reviewers**:

Reviewer #1: Thanks to the authors for addressing all my previous comments well. I don't have any further issues to raise.

THANKS

Reviewer #2: Thanks to the authors for their work in addressing all feedback! I think the authors have adequately addressed my comments, and have explained well where they disagreed. I find the findings of the paper are much easier to follow now and clarity has greatly improved. I have no further comments.

THANKS

Reviewer #3: Thank you for addressing my comments - I am satisfied with your responses. Congratulations on the study!

THANKS

**TECHNICAL EDITOR**

Ref: Rapid molecular testing or chest-X-ray or tuberculin skin testing for household contact assessment of tuberculosis infection: Results of a cluster-randomized trial

PMEDICINE-D-24-03440R2

Dear Menzies,

Thank you for your patience while we performed technical checks on your submission. Please see below for a list of requirements for you to address while conducting your revisions.

Journal Requirements:

1) Please ensure your short title is correct in the submission form, including symbols, italicisation etc. Your short title will be typeset from the submission form directly.

NOTE: Authors should provide a Short Title, around 70 characters long, that states the topic of the study.

For clinical trials, systematic reviews, or meta-analyses, the subtitle should include the study design.

SHORT TITLE: A randomized trial of three approaches for household contact investigation. (76 characters)

2) Please amend your detailed Financial Disclosure statement. This is published with the article. It must therefore be completed in full sentences and contain the exact wording you wish to be published.

1. State the initials, alongside each funding source, of each author to receive each grant.

2. State what role the funders took in the study. If the funders had no role in your study, please state: “The funders had no role in study design, data collection and analysis, decision to publish, or preparation of the manuscript.”

3. If any authors received a salary from any of your funders, please state which authors and which funders.

If you did not receive any funding for this study, please simply state: “The authors received no specific funding for this work.

Current statement (pasted below) complies with all above. Please advise if not.

*The study was supported by operating grants from the Canadian Institutes of Health Research (FDN-143350 to DM) and the Brazilian Ministry of Health (parecer técnico 3/2021, NUP 25000.012788/2021-61 to AT). Study authors were also supported by salary awards from Canada Research Chair award to DM, Conselho Nacional de Desenvolvimento Científico e Tecnológico of Brazil to AT and MCS. The funders had no role in the study design, data collection and analysis, decision to publish, or preparation of the manuscript.*

3) Thank you for providing your ethics statement "The study was approved by the Research Ethic Board of the Research Institute of the McGill University Health Centre on August 16, 2019 (Study 2020-5634 Form F1-59915), by the Brazilian National Ethical Board CONEP on May 7, 2020 (#4.014.402) and by the “Comité Local d’Éthique Pour la Recherche Biomédicale (CLERB) de l’Université de Parakou”, Benin, on December 30, 2019 (#0293). All HHC provided signed *written* informed consent. In Brazil, index TB patients also provided signed *written* informed consent."

Please state - Please state whether the consent was verbal/written.

OK – added ‘written” to ‘signed informed consent’, although I think “‘signed informed consent” covers it and adding ‘written’ seems un-grammatical....

4) Thank you for stating " The data from this study will be available from a public repository. "

In your Data Availability Statement, please ensure you have provided all necessary access information in complete sentences. Access information should include the name of any data repositories used as well as digital object identifiers (DOIs), accession numbers or codes, or other persistent identifiers for all relevant data.

See above. This is taking time to arrange. We are corresponding with C-PATH...

5) We have noticed that you have uploaded " GXT CONSORT Checklist_30Jan2025.docx, GXT Protocol_2019-06-03_Version 1_R1_REBapproved.docx, Statistical Analysis Plan GXT Jan2024 V7.docx " as Supporting Information files, but you have not included a list of legends. Please add a full list of legends for your Supporting Information files after the references list.

I think it fits better to add these to the list of supplemental materials ( figure and tables), so this was added at end of that section (Lines 600-603):

***Additional Supplemental materials:***

*GXT CONSORT Checklist_30Jan2025.docx,*

*GXT Protocol_2019-06-03_Version 1_R1_REBapproved.docx,*

*Statistical Analysis Plan GXT Jan2024 V7.docx*

If you have any questions or require assistance, please don't hesitate to get in touch via return email. To help us minimize inbox volumes, we kindly ask that you not reply to this email simply to confirm you have resubmitted your revisions.

Kind regards,

Anitha Samidurai

Publications Assistant

PLOS Medicine

[plosmedicine@plos.org](mailto:plosmedicine@plos.org)
